# The E3 ubiquitin ligase MARCHF8 restricts HSV-1 infection by inhibiting replication of the viral genome

**DOI:** 10.1016/j.jbc.2025.110567

**Published:** 2025-08-06

**Authors:** Yongyan Xia, L. Rayburn Nigos, Fernando Villalón-Letelier, Melkamu B. Tessema, Andrew G. Brooks, Eva Bartok, Rayk Behrendt, Sarah L. Londrigan, Patrick C. Reading, Rubaiyea Farrukee

**Affiliations:** 1 Department of Microbiology and Immunology, The University of Melbourne, at the Peter Doherty Institute for Infection and Immunity, Victoria, Australia; 2 Institute of Experimental Haematology and Transfusion Medicine, University Hospital, University of Bonn, Bonn, Germany; 3 The Institute of Clinical Chemistry and Clinical Pharmacology, University of Bonn, Bonn, Germany; 4 WHO Collaborating Centre for Reference and Research on Influenza, Victorian Infectious Diseases Reference Laboratory, The Peter Doherty Institute for Infection and Immunity, Victoria, Australia

**Keywords:** virology, herpesvirus, MARCHF8 protein, E3 ubiquitin ligase, innate immunity, virus restriction, viral immunology

## Abstract

Host cell restriction factors are intracellular proteins that target and inhibit virus replication. Membrane-associated RING-CH finger (MARCHF) proteins are a family of intracellular E3-ubiquitin ligases and some, including MARCHF8, have been implicated in restricting the replication of diverse RNA viruses. However, little is currently known as to whether MARCHF proteins mediate antiviral activity against DNA viruses. Herein, we used a doxycycline-inducible overexpression system to demonstrate that human MARCHF1 and MARCHF8 potently restrict productive HSV-1 replication and assessed MARCHF8-mediated restriction of HSV-1 in detail. A functional RING-CH domain and a tyrosine-based motif, located in the N- and C-terminal cytoplasmic tail of MARCHF8, respectively, were required for HSV-1 restriction. For many RNA viruses, MARCHF8-mediated restriction has been associated with downregulation of viral envelope glycoproteins from the cell surface, thereby limiting their subsequent incorporation into nascent virions. However, while MARCHF8 expression did not affect virus entry, translocation of viral genome to the nucleus and immediate early (IE) gene expression, it did inhibit HSV-1 genomic replication, and therefore subsequent late gene expression and release of infectious virions. MARCHF8-mediated restriction of HSV-1 occurred independent of other cellular factors known to impact genomic replication of HSV-1, namely SAMHD1 and CD81, and could also proceed efficiently in cells expressing a functional cGAS-STING pathway. To our knowledge, these studies are the first to demonstrate MARCHF8-mediated restriction against a human DNA virus. Moreover, inhibition of HSV-1 genomic replication represents a novel mechanism of MARCHF8-mediated virus restriction that is distinct to its reported antiviral activity against different RNA viruses.

Mammalian membrane-associated RING-CH-type finger (MARCHF) proteins were first identified as mammalian structural homologs of the viral membrane ubiquitin ligases, K3 and K5, which are expressed by Kaposi’s sarcoma-associated herpesvirus (KSHV) (1). The first mammalian MARCHF protein to be characterized was MARCHF8 (previously known as cellular modulator of immune regulation (c-MIR)), which is a functional homologue of the herpesvirus proteins MIR1 and MIR2, and shares similar substrate specificity (2). To date, 11 human MARCHF proteins have been reported. Most MARCHF proteins consist of an N-terminal RING-CH domain and two or more transmembrane domains, although MARCHF7 and MARCHF10 lack predicted transmembrane domains (reviewed in (3)). MARCHF proteins function as E3 ubiquitin ligases and catalyze the transfer of ubiquitin from ubiquitin conjugating enzymes (E2s) to target proteins, marking them for proteasomal and/or lysosomal degradation. Some MARCHF proteins are also known to auto-ubiquitinate to maintain homeostasis (4, 5, 6).

MARCHF proteins are expressed in a wide variety of cells and play significant roles in immune regulation, largely *via* modulation of immune molecules through ubiquitination. MARCHF1 and MARCHF8 have been particularly well studied, sharing approximately 60% sequence homology (7) as well as some overlapping substrate specificity. For example, both target and regulate the expression of major histocompatibility complex class II (MHC II) proteins and the costimulatory molecule cluster of differentiation (CD)86. While earlier studies reported selective recognition of other cell surface proteins such as CD44 and CD81 by MARCHF8, but not MARCHF1 (8, 9) we recently confirmed that one of the two MARCHF1 isoforms expressed does modulate CD44 and CD81 expression (10). MARHCHF1 is primarily expressed in secondary lymphoid tissue (reviewed in (3)) whereas MARCHF8 is expressed in a variety of cell types, including in terminally-differentiated myeloid cells such as monocyte-derived macrophages and dendritic cells (DC), and in a variety of other cell types, including T cells, epithelial cells and neurons (11, 12).

Emerging evidence indicates that certain MARCHF proteins modulate a range of different virus infections, either by regulating innate immune signaling or *via* direct interactions with specific viral components. For example, during influenza A virus (IAV) or vesicular stomatitis virus (VSV) infections, MARCHF5 can target and ubiquitinate MAVS aggregates to suppress MAVS-mediated type I IFN signaling and excessive immunopathology (13). However, MARCHF5 also mediates K48-linked polyubiquitination and degradation of RIG-I (14), highlighting its dual action in upregulating, as well as preventing, continued RIG-I-mediated signalling. Recent studies reported that MARCHF8 can target the cytoplasmic DNA sensor cyclic GMP-AMP synthase (cGAS), resulting in its ubiquitination and subsequent degradation (15). MARCHF8 can also be recruited by tetherin, a cellular protein with broad spectrum antiviral activity, to mediate downregulation of the MAVS signaling pathway *via* K27-linked ubiquitination of MAVS (16).

MARCHF proteins have also been shown to mediate direct antiviral activity against different RNA viruses. MARCHF8 downregulates the cell-surface expression of viral envelope proteins from a number of RNA viruses, including human immunodeficiency virus (HIV)-1, vesicular stomatitis virus (VSV), and murine leukemia virus (MLV) (17, 18, 19), thereby reducing their incorporation into nascent virions released from virus-infected cells. For VSV-G (18) and MLV p15E (19), this occurs by MARCHF8-mediated ubiquitination of lysine (K) residues in the cytoplasmic tail (CT) of each protein. Utilizing pseudovirus assays, K residues in the CT of envelope glycoproteins derived from other viruses, including members of the rhabdovirus, arenavirus, and togavirus families, were also shown to be critical for targeting by MARCHF8 (17, 20). For IAV, MARCHF8 was reported to polyubiquitinate a cytoplasmic K residue of the viral matrix 2 (M2) protein, redirecting it from the plasma membrane to lysosomal degradation (21). However, viruses pseudotyped with mutants of the HIV-1 envelope (Env) glycoprotein, including mutants in which cytoplasmic K residues were mutated (22) or the entire CT was deleted (23), were still effectively downregulated, indicating that MARCHF8 likely targets other viral and/or cellular proteins to mediate this effect. Similarly, pseudoviruses expressing mutants of Ebola virus glycoprotein (EboV-GP) or the severe acute respiratory syndrome coronavirus (SARS-CoV)-2 spike (S) glycoprotein lacking their respective CTs were also effectively downregulated by MARCHF8 (23). MARCHF1 and MARCHF2 have also been reported to downregulate cell-surface expression of HIV-1 Env (24).

Given its established role in regulating immune markers and its broad-spectrum antiviral activity against RNA viruses, we investigated the ability of MARCHF8 to restrict herpes simplex virus (HSV)-1, a human DNA virus from the *Ηerpesviridae* family. Herein, we demonstrate that inducible expression of MARCHF8 in human epithelial cell lines (HEK293T, HeLa, BEAS-2B) and monocytic tumor cell line (THP-1) resulted in potent inhibition of productive replication of both HSV-1 and HSV-2. MARCHF8 did not inhibit virus entry and translocation of viral genome to the nucleus but did inhibit HSV-1 genomic replication and late gene expression. These findings indicate that MARCHF8 inhibits productive HSV-1/2 replication *via* a mechanism that is distinct from its reported antiviral activity against RNA viruses.

## Results

### Inducible expression of MARCHF8 in human epithelial cells inhibits productive HSV-1 replication

**Figure 1:**
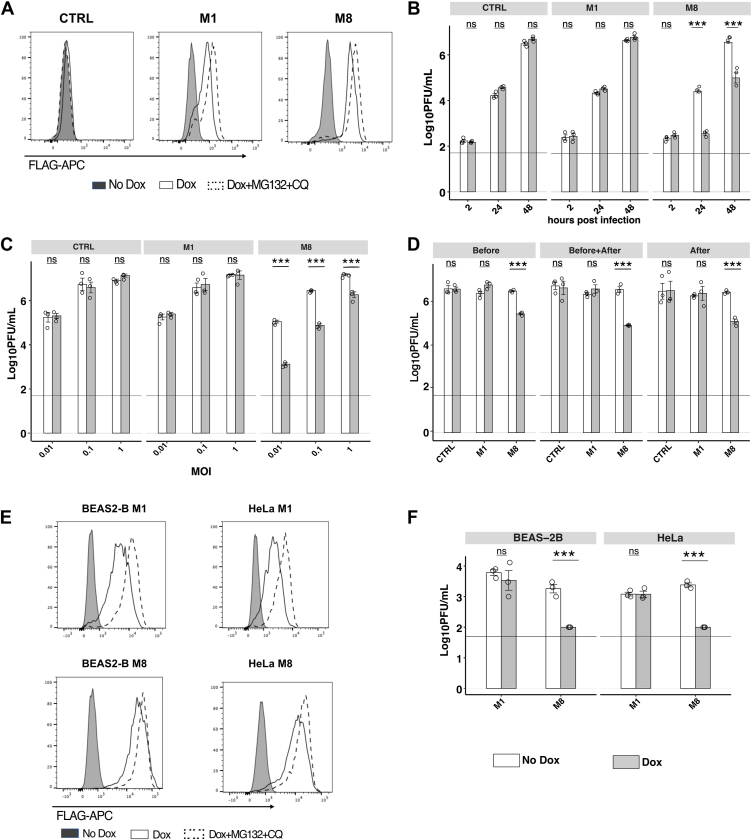
**Inducible expression of MARCHF8 in human epithelial cells inhibits productive replication of HSV-1**. *A*, 293T cells with doxycycline (DOX)-inducible expression of FLAG-tagged MARCHF1 (M1) or MARCHF8 (M8), or of an irrelevant intracellular control protein lacking a FLAG tag (CTRL) were cultured in the presence (Dox) or absence (No Dox) of 1 μg/ml Dox for 24 h (*solid line*), or in the presence of Dox for 20 h before addition of MG132 and chloroquine (MG132+CQ) for the last 4 h. Cells were then fixed and stained for intracellular expression of FLAG-tagged proteins and analysed by flow cytometry. Representative histograms are shown. *B* and *C*, 293T cells with Dox-inducible protein expression were incubated in the presence (Dox, *grey bars*) or absence (No Dox, *white bars*) of Dox for 24 h and then (*B*) infected with the KOS strain of HSV-1 (MOI 0.1) and supernatants collected at 2, 24 and 48 h post-infection (hpi), or (*C*) infected with KOS strain of HSV-1 at different MOIs (0.01, 0.1 and 1) and supernatants collected at 48 hpi. Virus titres in cell-free supernatants were determined by plaque assay on Vero cells. Representative technical replicates from one of *B*) n = 2 or *C*) n = 3 independent experiments shown. *D*, 293T cells with Dox-inducible protein expression were incubated with 1ug/ml Dox for 24 h before infection (Before), both before and after infection (Before + After) or Dox was added only after infection (After). Cells were infected with HSV-1 (MOI 0.1) and virus titres in clarified supernatants collected at 48 hpi were determined by plaque assay. Representative technical replicates from one of n = 3 independent experiments are shown. *E*, BEAS2B or HeLa cells with Dox-inducible expression of FLAG-tagged M1 or M8 were cultured in the presence (Dox) or absence (No Dox) of 1 ug/ml Dox for 24 h (*solid line*), or in the presence of Dox for 20 h before addition of MG132 and chloroquine (MG132+CQ) for the last 4 h. Cells were then fixed and stained for intracellular expression of FLAG-tagged proteins and analysed by flow cytometry. Representative histograms are shown. *F*, BEAS2-B or HeLa cells with Dox-inducible protein expression were incubated in the presence (Dox, *grey bars*) or absence (No Dox, *white bars*) of Dox for 24 h, infected with HSV-1 (MOI = 0.1) and virus titres in clarified supernatants determined at 48 hpi. Technical replicates from one of two independent experiments are shown. Limit of detection for plaque assay results are shown as a *horizontal line*. Statistical analysis was performed using a mixed effects model utilizing data points from all experiments, as described in Experimental procedures. ∗*p* < 0.01, ∗∗*p* < 0.001, ∗∗∗*p* < 0.0001 and ns = not significant.

MARCHF proteins, particularly MARCHF8 (M8), have been shown to act as potent restriction factors against diverse RNA viruses; however, little is known regarding their antiviral activity against DNA viruses. In a recent study, we used cell lines with doxycycline (Dox)-inducible protein expression to demonstrate that M8, but not the closely related MARCHF1 (M1, isoform 1.2), restricted IAV at a late stage in the virus replication cycle (25). To assess the ability of M1 and M8 to inhibit HSV-1, we first used flow cytometry to confirm Dox-inducible expression of FLAG-tagged M1 and M8 proteins in 293T cells (Fig. 1*A*), noting that expression of either protein was enhanced in the presence of MG132 and chloroquine (CQ), to inhibit proteasomal and lysosomal degradation, respectively. A control (CTRL) cell line with Dox-inducible expression of cytoplasmic hen egg ovalbumin and lacking a N-terminal FLAG tag was included for comparison. In previous studies, we demonstrated that inducible M1 and M8 both effectively downregulated cell-surface CD86 expression (10, 25), confirming functionality of both FLAG-tagged proteins.

To determine if M1 or M8 could restrict HSV-1 growth, cells cultured in the presence or absence of Dox were infected with HSV-1 (strain KOS, MOI = 0.1) and titers of infectious virus in clarified supernatants were determined at 2, 24, and 48 h post-infection (hpi). Relative to 2 hpi, virus titers increased progressively at 24 and 48 hpi in all cell lines tested (Fig. 1*B*). While we did not observe significant differences in titers of HSV-1 recovered from CTRL or M1 cells in the presence or absence of Dox, inducible expression of M8 resulted in significantly reduced titers of HSV-1 (*p* < 0.001) at 24 and 48 hpi. Similarly, following infection of cells with increasing MOI, we observed that cells expressing M8, but not M1, restricted HSV-1 growth at all MOI tested (Fig. 1*C*). In all experiments to date, 293T cells were cultured in the presence or absence of Dox for 24 h prior to and at all time points following inoculation with HSV-1. Next, we investigated the potency of M8-mediated restriction of HSV-1 if Dox was added prior to (Before), before and after (Before + After), or only after (After) inoculation with HSV-1. As seen (Fig. 1*D*), inducible M8, but not M1, restricted HSV-1 growth under all experimental conditions, noting that restriction was most potent when Dox was added before and after inoculation with HSV-1. Therefore, cells were cultured in Dox prior to and after HSV-1 in all subsequent experiments.

In a recent study, we reported that some restriction factors mediate antiviral activity in a cell type-specific manner (26). Therefore, we generated human epithelial cell lines (HeLa and BEAS-2B) with Dox-inducible expression of M1 and M8 and confirmed intracellular expression of FLAG-tagged proteins by flow cytometry (Fig. 1*E*), as well as the ability of M8, but not M1, to mediate potent anti-HSV-1 activity (Fig. 1*F*).

We also analyzed whether levels of inducible M1/8 were modulated during HSV-1 infection. For these experiments, 293T, BEAS-2B, or HeLa cells were infected with HSV-1 TK GFP (MOI 2), and at 8 hpi we used flow cytometry to compare levels of FLAG-tagged M1/M8 protein in GFP^+^ (HSV-1-infected) cells vs uninfected (mock) cells (Fig. S1*A*; gating strategy). Preliminary experiments examining later time points showed high levels of cell death at 24 or 48 hpi at >50% or >70% total cells, respectively, even when using low MOI conditions. As seen (Fig. S1*B*), levels of inducible M1 and M8 were significantly reduced in HSV-1-infected cells (indicated by both a reduction in % of FLAG^+^ cells and gMFI of FLAG^+^ cells). Moreover, we showed that simultaneous blocking of proteasomal and lysosomal protein degradation using MG132/CQ recovered a high degree of M1/M8 expression (Fig. S1*C*). Together, our results indicate that HSV-1 infection can reduce the expression of inducible M1 and M8, noting that the impact on M1 expression was more marked in the cell types examined.

### A functional RING-CH domain is essential for MARCHF8-mediated restriction of HSV-1 and of HSV-2

**Figure 2:**
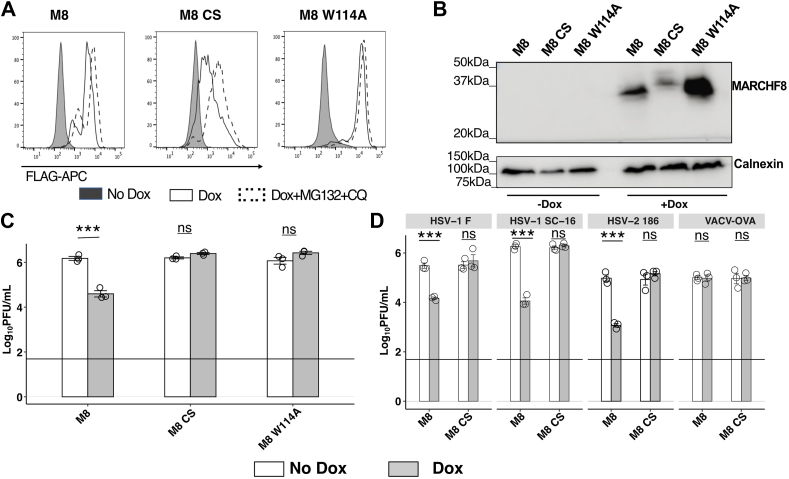
**A functional E3 ligase domain is required for antiviral activity against a-herpesviruses.***A* and *B*, 293T cells with Dox-inducible expression of FLAG-tagged parental M8 or E3 ligase mutants (M8 CS or M8 W114A) were cultured in the presence or absence of Dox for 24 h or in Dox for 20 h before addition of MG132+CQ for the last 4 h. Dox-inducible protein expression was then determined (*A*) by flow cytometry using an anti-FLAG mAb to stain for intracellular protein expression, or (*B*) by Western blot using a M8-specific polyclonal Ab, with a Calnexin loading control. *C* and *D*, 293T cells with Dox-inducible protein expression were cultured in the presence (Dox) or absence (No Dox) of Dox and then infected with (*C*) HSV-1 KOS (MOI 0.1), or (*D*) HSV-1 strains F or SC-16, HSV-2 strain 186 or VACV-OVA (all at MOI 0.1). Virus titres in clarified supernatants collected at 48 hpi were determined by plaque assay. Technical replicates from one of three independent experiments are shown. Limit of detection for plaque assay results are shown as a *horizontal line*. Statistical analysis was performed using a mixed effects model utilizing data points from all experiments, as described in Experimental procedures. ∗*p* < 0.01, ∗∗*p* < 0.001, ∗∗∗*p* < 0.0001 and ns = not significant.

**Figure 3:**
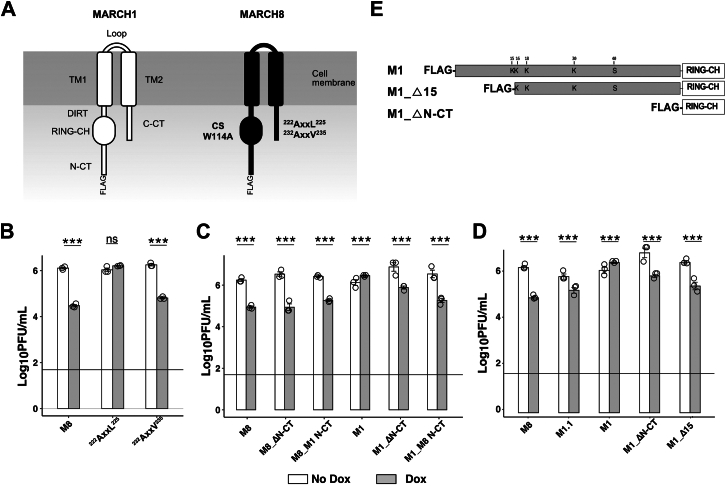
**Structural characteristics of MARCHF8 and MARCHF1 that contribute to their ability to inhibit productive replication of HSV-1.***A*, schematic showing the domain structure of M8 and highlight M8 mutants utilized in this study. *B* and *C*, stable 293T cell lines with DOX-inducible expression of parental M8 or M1 or (*B*) M8 C-CT tyrosine motif mutants M8-^222^Axx^L225^ or M8-^232^AxxV^235^, (*C*) M8 with N-CT deletion (M8_ΔN-CT) or substitution for the N-CT of M1 (M8_M1 N-CT), or M1 with N-CT deletion (M1_ΔN-CT) or substitution for the N-CT of M8 (M1_M8 N-CT) were generated and characterized previously (25). *D/E*, schematic of M1 isoform 2 (M1) showing specific lysine (K) residues in the N-CT, as well as M1 N-CT deletion mutants lacking the entire N-CT (M1_ΔN-CT) or lacking 15 residues from the N-CT of M1.2 (M1_Δ15). 293T cells with DOX-inducible expression of M1, M1 isoform 1 (M1.1) and M1_Δ15 were generated and characterized previously (10). *B*, *C*, and *E*, cells were cultured in the presence (Dox) or absence (No Dox) of 1 μg/ml Dox and then infected with HSV-1 KOS (MOI 0.1). Virus titres in clarified supernatants collected at 48 hpi were determined by plaque assay. Technical replicates from 1 of (*A*) n = 3, (*B*) n = 5, and (*C*) n = 5 independent experiments are shown. Limit of detection for plaque assay results are shown as a *horizontal line*. Statistical analysis was performed using a mixed effects model utilizing data points from all experiments as described in Experimental Procedures. ∗*p* < 0.01, ∗∗*p* < 0.001, ∗∗∗*p* < 0.0001 and ns = not significant.

Mutations in the RING domain are known to disrupt E3 ligase activity and have been shown to abrogate the ability of M8 to reduce expression of viral envelope glycoproteins and/or the infectivity of RNA viruses (11, 22, 25, 27). Therefore, flow cytometry (Fig. 2*A*) and Western blot (Fig. 2*B*) were used to confirm Dox-inducible expression of FLAG-tagged parental M8, as well as the E3 ligase mutants M8-CS or M8-W114A (Fig. 3*A*), in 293T cells. Moreover, potent restriction of HSV-1 was observed in cells expressing M8, but this was completely abrogated in cells expressing either M8-CS or M8-W114A mutants (Fig. 2*C*). These data confirm the critical importance of a functional RING-CH domain for M8-mediated restriction of HSV-1. As observed for M8, expression levels of M8 E3 ligase mutant proteins were also reduced in HSV-1-infected cells (Fig. S2*A*), noting that the reduction in M8-W114A expression was relatively modest.

Next, we assessed the ability of cells with inducible M8 or M8-CS expression to inhibit the growth of different strains of HSV-1, as well as HSV-2. In addition to the HSV-1 strain KOS (Fig. 1*D*), M8 also potently restricted the growth of F and SC-16 strains of HSV-1, as well as HSV-2 strain 186 (Fig. 2*D*), whereas cells expressing M8-CS did not. Of interest, M8 did not restrict the growth of a vaccinia virus (Fig. 2*D*), an unrelated DNA virus that replicates within the cytoplasm of infected cells (28). To ensure results were not due to lower expression of M8-CS (Fig. 2*B*), we also confirmed loss of virus restriction using the M8-W114A mutant following infection with HSV-1 and HSV-2 (Fig. S2*B*). Of note, a small but significant enhancement of virus titers was observed in DOX-treated M8-W114A cells in some experiments.

### Structural characteristics of MARCHF8 and MARCHF1 that contribute to their ability to restrict HSV-1

When considering the C-terminal cytoplasmic tail (C-CT) of M8, the ^222^YxxL^225^, but not the ^232^YxxV^235^ motif, was reported to be critical for M8-mediated restriction of HIV-1 (22). We previously generated 293T cells with Dox-inducible expression of M8 C-CT tyrosine mutants (^222^AxxL^225^ or ^232^AxxV^235^) (Fig. 3*A*) to demonstrate (i) inducible expression of each FLAG-tagged mutant, as well as the ability of both mutants to effectively downregulate CD86, by flow cytometry, and (ii) the importance of the ^222^YxxL^225^ motif for M8-mediated restriction of IAV (25). We used the same cell lines to investigate the importance of tyrosine-based motifs in the M8 C-CT in contributing to the restriction of HSV-1. Following infection, parental cells expressing M8 and the M8 ^232^AxxV^235^ mutant restricted the growth of HSV-1, whereas M8 ^222^AxxL^225^ did not (Fig. 3*B*). Thus, as for HIV-1 (22) and IAV (25), mutation of the ^222^YxxL^225^ motif in the M8 C-CT abrogated its ability to inhibit productive HSV-1 replication.

M1 and M8 show a high degree of sequence conservation across the entire protein, with the N-terminal cytoplasmic domain (N-CT) showing the greatest degree of variability between the two proteins (29). In a previous study, we generated cell lines expressing M1 and M8 with their N-CT deleted (M1_ΔN-CT and M8_ΔΝ-CT, respectively), as well as chimeric mutants where the N-CT of M8 was swapped onto M1 (M1_M8 N-CT), and where the N-CT of M8 was swapped onto M1 [M1_M8 N-CT]) (25). These studies confirmed expression of each FLAG-tagged deletion or chimeric protein and confirmed no major differences in their ability to downregulate CD86 as a measure of functionality (25). We next used these cell lines to investigate the ability of the N-CT of M1 and M8 to modulate antiviral activity against HSV-1. When considering M8, cell lines expressing a deletion mutant lacking the M8 N-CT (M8_ΔΝ-CT) or a chimeric mutant of M8 expressing the M1 N-CT (M8_M1 N-CT) both inhibited HSV-1 to significant levels (Fig. 3*C*). Moreover, deletion of the M1 N-CT (M1_ΔΝ-CT) resulted in acquisition of HSV-1 restriction and a chimeric mutant of M1 expressing the M8 N-CT (M1_M8 N-CT) also potently inhibited HSV-1 growth (Fig. 3*D*). Similar to previous results reported herein, DOX-inducible M1 did not restrict HSV-1 growth, although in this case a modest, but significant enhancement in virus titers was observed across multiple experiments. These findings indicate that (i) the N-CT of M8 is not necessary for M8-mediated restriction of HSV-1, whereas in contrast (ii) the N-CT of M1 in fact inhibits the anti-HSV-1 activity of the rest of the protein, and (iii) anti-HSV-1 activity can be restored to M1 by deletion of N-CT or by substitution of the M8-N-CT into M1.

Two isoforms of M1 (M1.1 and M1.2) have been described which are very similar in overall structure and homology but show major differences in the sequence of their N-CT domain (10) and differing expression levels in human peripheral blood mononuclear cells and macrophages (10) We recently confirmed that Dox-inducible expression of either M1.1 or M1.2 in 293T cells resulted in effective downregulation of cell-surface CD86; however, only M1.1 also downregulated cell-surface CD44 and CD81 (10). Furthermore, we showed that M1.1, but not M1.2, restricted IAV growth and generated deletion mutants to identify the residues in the M1.2 N-CT responsible for blocking its anti-IAV activity (10). For experiments presented to date in the current study, all M1 cell lines have expressed the M1.2 isoform. In previous studies, we confirmed that M8, but not M1.2, mediated potent anti-IAV activity (25) and later reported that the M1.1 isoform did indeed restrict IAV replication (10). Therefore, we next compared the ability of M1.1 *versus* M1.2 to restrict growth of HSV-1. As seen (Fig. 3*D/E*), infection of 293T cells with Dox-inducible M1.1 resulted in potent inhibition of HSV-1 growth whereas cells expressing M1.2 (shown as M1 in figure) did not.

Given that autoubiquitination of M1 has been associated with regulating its own intracellular expression levels (4), we previously generated mutants with stepwise deletions to remove specific lysine (K) residues from the N-CT of M1.2 (at residues 15, 16, 29 and 40, which might serve as potential targets for autoubiquitination), generated stable 293T cell lines with inducible expression of each mutant, and reported that deletion of 16 residues from the M1.2 N-CT was sufficient to restore its antiviral activity against IAV (10). When using these cell lines to assess HSV-1 growth (Fig. 3*E*), deletion mutants lacking 16, 29, and 40 residues from the N-CT of M1.2 all mediated potent anti-HSV-1 activity (data not shown), however even deletion of 15 residues from the N-CT (M1_Δ15) was sufficient to restore the anti-HSV-1 activity of M1.2 (Fig. 3*E*). Therefore, deletion of the first 15 N-terminal residues of the M1.2 N-CT domain is sufficient to restore its ability to restrict HSV-1 growth.

Finally, we show that expression of all M1/M8 deletion mutants and chimeric proteins was significantly reduced in HSV-1-infected compared to mock-infected cells (Fig. S3). Despite this, we observed clear evidence that all could restrict HSV-1 replication to a significant degree, except inducible M1.

### MARCHF8 does not inhibit HSV-1 entry and translocation to the nucleus but does inhibit HSV-1 genomic replication and late viral gene expression

**Figure 4:**
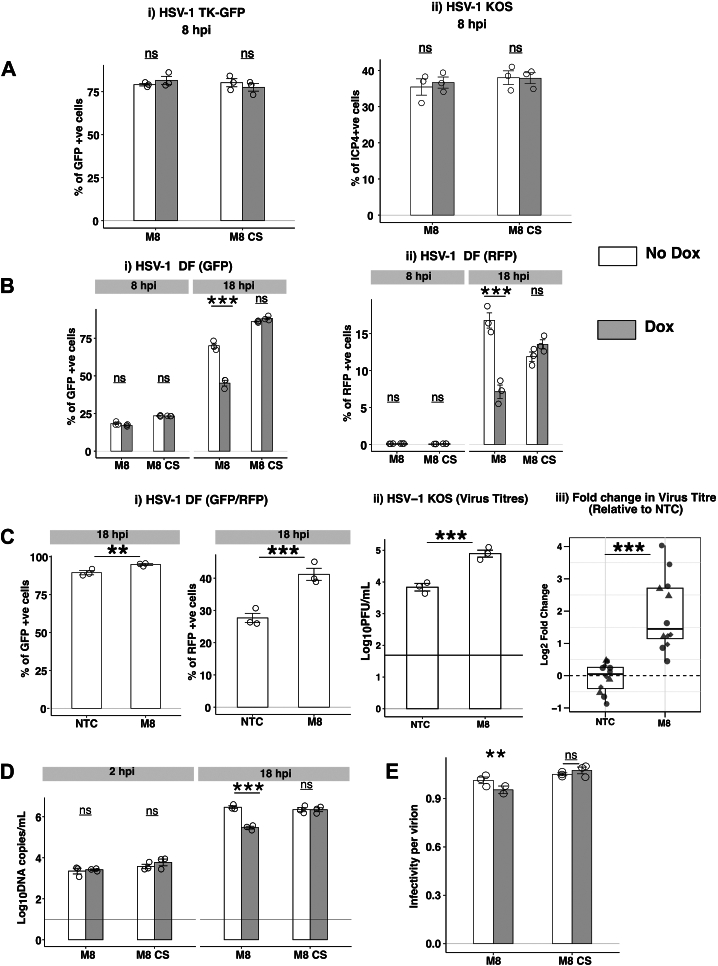
**MARCHF8 does not inhibit HSV-1 entry and translocation to the nucleus but does inhibit HSV-1 genomic replication and late viral gene expression.***A*, 293T cells with Dox-inducible expression of FLAG-tagged M8 or M8-CS were cultured in the presence (Dox) or absence (No Dox) of 1ug/ml Dox for 24 h (*solid line*) and then infected with i) HSV-1 KOS-TK-GFP (MOI 2) or ii) HSV-1 KOS (MOI 4) and analysed at 8 hpi. (i) Cells were fixed and the percentage of GFP^+^ cells was determined by flow cytometry. (ii) Cells were fixed, permeabilized and stained with a mAb to the immediate early HSV-1 protein ICP4, followed by Alexa Fluor 488-conjugated chicken anti-mouse Ig (*right panel*). Representative technical replicates from (*A*) n = 3 and (*B*) n = 2 experiments are shown. *B*, following 24 h culture in the presence or absence of Dox, cells were infected with a double fluorescent HSV-1 virus (DF-HSV-1), expressing GFP and RFP under control of the HSV-1 gB or gC promoters, respectively. At either 8 (MOI = 2) or 18 (MOI = 5) hpi, cells were fixed and EGFP and RFP expression analysed by flow cytometry. Technical replicates from one of three independent experiments are shown. *C*, (i) A549 cells were transfected with 10 μM of M8-specific or NTC siRNA for 48 h, infected with DF-HSV-1 (MOI 1) and levels of GFP or RFP expression determined by flow cytometry at 18 hpi. Technical replicates from one of four independent experiments are shown. (ii) A549 cells were transfected with 10 μM of M8-specific or NTC siRNA for 48 h and then infected with HSV-1 KOS (MOI 0.001). Virus titres in clarified supernatants collected at 36 hpi were determined by plaque assay. Technical replicates from one of four independent experiments are shown. Limit of detection for plaque assay results are shown as a horizontal line. (iii) Pooled data of log_2_ fold change in viral titres from average NTC titres across four experiments are shown, different symbol used for each experiment. *D*, 293T cells with Dox-inducible protein expression cultured for 24 h in the presence or absence of Dox were infected with HSV-1 (MOI 0.1). At 2 and 18 hpi, total cellular DNA was extracted for real-time qPCR. HSV-1 DNA copy number was determined relative to a standard curve generated using a plasmid expressing the HSV-1 TK gene. Technical replicates from one of five independent experiments are shown and the *horizontal line* indicates the limit of detection. *E*, infectivity per virion was determined as the ratio between the titre of infectious virus (Log_10_ PFU/ml) and the copy number of the Tk gene (Log_10_DNA copies/ml) from each sample. Technical replicates from one of three independent experiments are shown. Statistical analysis was performed using a mixed effects model utilizing data points from all experiments. ∗*p* < 0.01, ∗∗*p* < 0.001, ∗∗∗*p* < 0.0001 and ns = not significant.

Next, we aimed to determine which steps in the HSV-1 replication cycle were inhibited by M8. Initial studies used a recombinant HSV-1 virus with a disrupted thymidine kinase (TK) gene that expresses green fluorescent protein (GFP) under the HCMV promoter (HSV-1-TK-GFP) (30). When cells are infected with this virus, GFP expression occurs independently of HSV-1 genomic replication but requires entry of the viral genome into the nucleus. As such, this virus is useful to assess the impacts of M8 on the early stages of HSV-1 replication within infected cells. Therefore, M8 and M8-CS 293T cells cultured in the presence or absence of Dox were infected with HSV-1-TK-GFP (MOI = 10) and the percentage of GFP^+^ cells determined at 8 hpi. As shown (Figs. 4*A*(i) and S4*A*), no significant differences were observed in percentage of GFP^+^ cells in the presence or absence of inducible M8, indicating that M8 does not inhibit cellular entry of HSV-1 or translocation of genomic HSV-1 DNA from the cytoplasm into the nucleus. As an independent approach, M8 and M8-CS cells cultured in the presence or absence of Dox were also infected with the HSV-1 KOS strain and stained for intracellular expression of HSV-1 ICP4 protein, an immediate early (IE) gene that is expressed prior to genomic replication (31). Similar to results obtained using the HSV-1-TK-GFP virus, no significant differences were observed in the percentage of ICP4^+^ cells at 8 hpi in the presence or absence of Dox (Figs. 4*A*(ii) and S4*B*).

To assess the impact of M8 on HSV-1 late gene expression we used a recombinant HSV-1 virus that expresses GFP and red fluorescent protein (RFP) from the HSV-1 glycoprotein (g)B and gC promoters, respectively (HSV-1 double fluorescent (DF)). HSV-1 gB is a leaky-late (γ1) gene expressed prior to and after HSV-1 genomic replication (32). In contrast, HSV-1 gC is a true late (γ2) gene that requires HSV-1 genomic replication for expression (32). Thus, when HSV-1 genomic replication is blocked, early GFP expression will proceed, whereas RFP expression will be suppressed. Preliminary experiments used flow cytometry to confirm that (i) infected cells expressed GFP, but not RFP, at 8 hpi, (ii) cells expressed both GFP and RFP at 18 hpi, and (iii) that phosphonoacetic acid (PAA), a known inhibitor of HSV-1 replication (33), completely inhibited RFP expression at 18 hpi, although some expression of GFP was still observed (data not shown). These data validate the functionality of the HSV-1 DF virus to examine impacts on HSV-1 early gene expression (GFP at 8 hpi, prior to genomic replication) and late gene expression (RFP at 18 hpi, after genomic replication) and are consistent with published studies describing the generation and characterization of this virus (34).

Next, we investigated the impact of inducible M8 on HSV-1 early gene expression prior to genomic replication (gB-GFP, 8 hpi), as well as late gene expression following genomic replication (gC-RFP, 18 hpi). M8 and M8-CS cells were therefore cultured in the presence or absence of Dox, infected with HSV-1 DF and the percentage of GFP^+^ and RFP^+^ cells determined at 8 and 18 hpi. At 8 hpi, similar percentages of GFP^+^ cells were detected in M8 and M8-CS cells cultured the presence or absence of Dox, whereas RFP levels were very low (<0.5% RFP^+^ cells) (Figs. 4*B* and S5). At 18 hpi, HSV-1 infection in the presence of inducible M8, but not M8-CS, resulted in significantly reduced percentages of both GFP^+^ and RFP^+^ cells (Figs. 4*B* and S5). These findings suggest that M8 inhibits HSV-1 at a step after early HSV-1 gene expression and prior to late gene expression. Of note, as GFP is linked to leaky late gene expression, the significant reduction in GFP observed at 18 hpi is likely due to reduced GFP expression from the gB promoter following HSV-1 genomic replication.

To assess the impact of endogenous M8 on HSV-1 late gene expression, we utilized qPCR to screen cell lines (293T, A549 and THP-1, including PMA-differentiated THP-1) for levels of endogenous M8 expression. Amongst these, A549 cells showed the highest levels of endogenous M8 expression (Fig. S6*A*(i)). We then confirmed efficient siRNA-mediated knockdown of M8 in A549 cells relative to cells treated with non-targeting control (NTC) siRNA (Fig. S6*A*(ii)). Next, following siRNA-mediated knockdown of M8, A549 cells were infected with HSV-1-DF virus and expression of GFP and RFP (driven from the gB and gC promotors, respectively), were determined at 18 hpi. Relative to NTC-treated control cells, siRNA-mediated knockdown of endogenous M8 was associated with a significant enhancement in the percentage of GFP^+^ and RFP^+^ cells at 18 hpi (Figs. 4*C*(i) and S7), consistent with inhibition of HSV-1 late gene expression by endogenous M8. Moreover, following siRNA-mediated knockdown of M8, A549 cells were infected with HSV-1 KO (MOI 0.001) at virus titres determined at 36 hpi. Relative to NTC-treated cells, knockdown of endogenous M8 significantly enhanced virus titers (Fig. 4*C*(ii-iii)), consistent with the ability of endogenous M8 to restrict HSV-1 replication. While DOX-inducible M8 expression in 293T cells is ∼30-fold higher than expression of endogenous M8 in A549 cells by qPCR (data not shown), these data confirm that like inducible M8, endogenous M8 can also inhibit HSV-1 replication. Of note, we also demonstrated that HSV-1 infection had minimal impact on the expression of endogenous M8 in 293T, A549 or THP-1 cells at the mRNA level, although we did observe a modest upregulation in expression of M1 mRNA following infection (Fig. S6*B*).

To determine whether M8 expression resulted in inhibition of HSV-1 genomic replication, cells (M8 and M8-CS 293T) cultured in the presence and absence of Dox were infected with HSV-1 (MOI 0.1) and HSV-1 genome copy numbers determined at 2 hpi (residual cell-associated virus prior to genomic replication) and 18 hpi (following infection and viral genomic replication). While no differences in genome copy number were observed between uninduced and Dox-induced cells at 2 hpi (Fig. 4*D*), expression of M8, but not M8-CS, resulted in a significant reduction in genome copy number at 18 hpi (Fig. 4*D*) confirming that M8 expression resulted in inhibition of HSV-1 genomic replication.

For enveloped RNA viruses such as HIV-1, reduced incorporation of viral envelope glycoproteins into nascent virions in the presence of M8 results in a reduced infectivity per particle of virions released from infected cells (11). Similar findings have been reported for IAV (25) and for pseudotyped viruses expressing VSV-G envelope glycoproteins (21). We used a similar approach to determine the ratio between titers of infectious HSV-1 (plaque assay) and the total number of particles released (qPCR) in cell-free supernatants from HSV-1-infected cells in the presence or absence of inducible M8 or M8-CS. While previous studies reported a substantial (>90%) reduction in infectivity per particle for HIV-1 (11) and IAV (25) in the presence of M8, this was not the case for HSV-1. Instead, we observed a very modest (<10%), reduction in the infectivity per particle of HSV-1 released from infected cells in the presence of inducible M8 (Fig. 4*E*), consistent with a block in HSV-1 replication resulting in reduced release of infectious virions rather than a reduced infectivity per particle of nascent virions.

### Inducible expression of MARCHF8 in PMA-differentiated THP-1 cells inhibits productive HSV-1 replication

**Figure 5:**
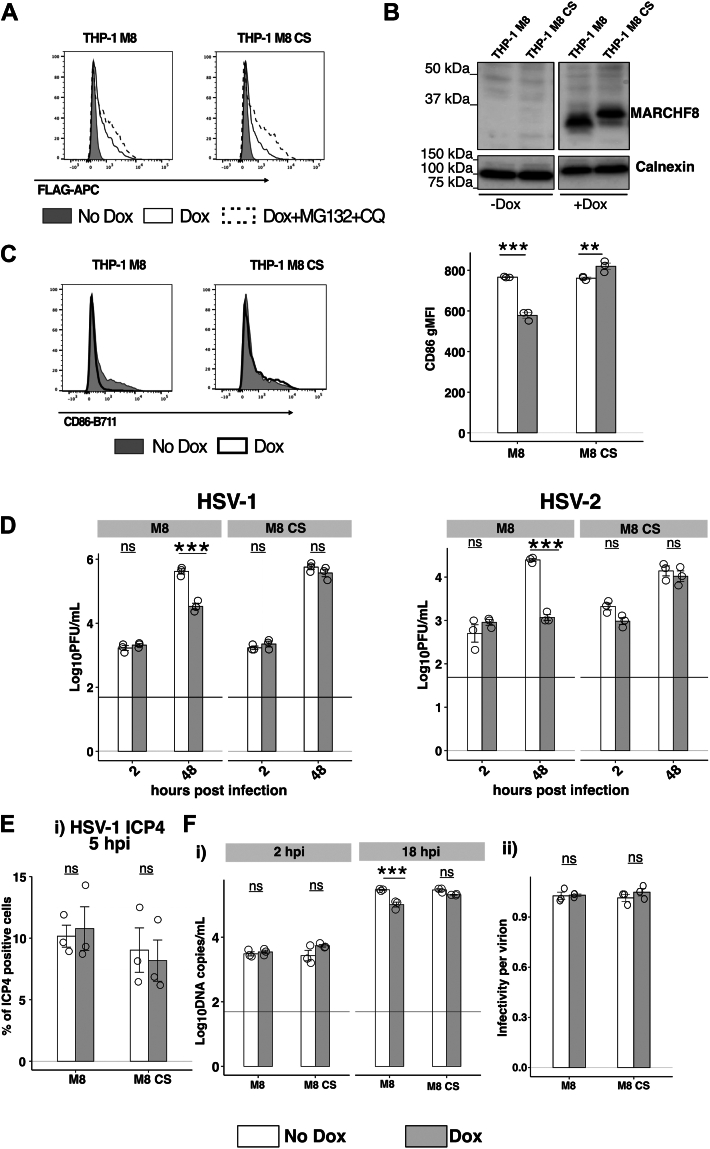
**Inducible expression of MARCHF8 in human macrophage-like THP-1 cells inhibits productive replication of HSV-1.***A* and *B*, PMA-differentiated THP-1 cells with Dox-inducible expression of FLAG-tagged M8 or M8-CS were cultured in No Dox, in Dox for 24 h or in Dox for 20 h before addition of MG132+CQ for the last 4 h. Dox-inducible protein expression was then determined (*A*) by flow cytometry using an anti-FLAG mAb to stain for intracellular protein expression, or (*B*) by Western blot using a M8-specific polyclonal Ab, with a Calnexin loading control. *C–F*, PMA-differentiated THP-1 cells were cultured 24 h in the presence (Dox) or absence (No Dox) of 1 μg/ml Dox. *C*, cells were collected and levels of cell surface CD86 were determined by flow cytometry (i) Representative histograms and (ii) geometric mean fluorescence intensity (gMFI) of CD86 from one of three independent experiments are shown. *D*, cells were then infected with HSV-1 KOS or HSV-2186 (both at MOI 5) and virus titres in clarified supernatants collected at 2 and 48 hpi were determined by plaque assay. Technical replicates from one of 5 (HSV-1) or one of 2 (HSV-2) independent experiments are shown. *E*, cells were then infected with HSV-1 KOS (MOI 10) and at 5 hpi cells were fixed, permeabilized and stained with a mAb to the immediate early HSV-1 protein ICP4, followed by Alexa Fluor 488-conjugated chicken anti-mouse Ig (*right panel*) and analysed by flow cytometry. Technical replicates from one of two independent experiments are shown. *F*, cells were then infected with HSV-1 KOS (MOI 1). At 2 and 18 hpi, total cellular DNA was extracted for real-time qPCR. (i) HSV-1 DNA copy number was determined relative to a standard curve generated using a plasmid expressing the HSV-1 TK gene. Technical replicates from one of three independent experiments are shown and the *horizontal line* indicates the limit of detection. (ii) Infectivity per virion was determined as the ratio between the titre of infectious virus (Log_10_ PFU/ml) and the copy number of the TK gene (Log_10_DNA copies/ml) from each sample. Statistical analysis was performed using a mixed effects model utilizing data points from all experiments. ∗*p* < 0.01, ∗∗*p* < 0.001, ∗∗∗*p* < 0.0001 and ns = not significant.

While epithelial cells represent a key target for HSV-1 infection, immune cells such as macrophages are also susceptible to infection and key restriction factors, such as SAMHD1, have been reported to exert anti-HSV-1 activity in macrophage-like cells (35). Therefore, THP-1 monocytic cells with Dox-inducible expression of M8 or M8-CS were generated, differentiated for 24 h in the presence of PMA and used to confirm expression of FLAG-tagged proteins by flow cytometry (Fig. 5*A*) and Western blot (Fig. 5*B*). Inducible expression of M8, but not M8-CS, also resulted in downregulation of endogenous cell surface CD86 confirming expression of functional M8 protein in THP-1 cells (Fig. 5*C*). Next, we confirmed the ability of M8, but not M8-CS, to inhibit growth of both HSV-1 and HSV-2 in PMA-differentiated THP-1 cells (Fig. 5*D*). M8-mediated inhibition of HSV-1 growth in THP-1 cells occurred at a similar step in the virus replication cycle to that described using 293T epithelial cells, as M8 expression in differentiated THP-1 did not inhibit the percentage of cells that stained positive for the IE gene product ICP4 (Figs. 5*E* and S4*C*) but did result in significant inhibition of HSV-1 genomic replication (Fig. 5*F*(i)). Moreover, expression of inducible M8 in PMA-differentiated THP-1 cells did not result in a significant reduction in the infectivity per virion of HSV-1 released from infected cells (Fig. 5*F*(ii)). These findings are distinct to the potent inhibition of infectivity per particle reported for HIV-1 (11), IAV (25) and other RNA viruses, which is associated with reduced incorporation of viral envelope glycoproteins into nascent virions.

### Cellular CD81 is not essential for MARCHF8-mediated restriction of HSV-1 replication in epithelial or monocytic cell lines

In neurons, CRISPR/Cas9-mediated knockout (KO) of CD81 did not impact HSV-1 IE gene expression but did inhibit HSV-1 genomic replication and subsequent release of infectious virions (36). Given that (i) we and others have demonstrated that inducible expression of M8 results in potent downregulation of CD81 (8, 10, 25), and (ii) M8-mediated restriction of HSV-1 also does not impact HSV-1 IE gene expression but does inhibit viral genomic replication, we investigated whether M8-mediated regulation of CD81 contributes to the mechanism through which M8 restricts HSV-1 growth in epithelial and/or macrophage cell lines.

**Figure 6:**
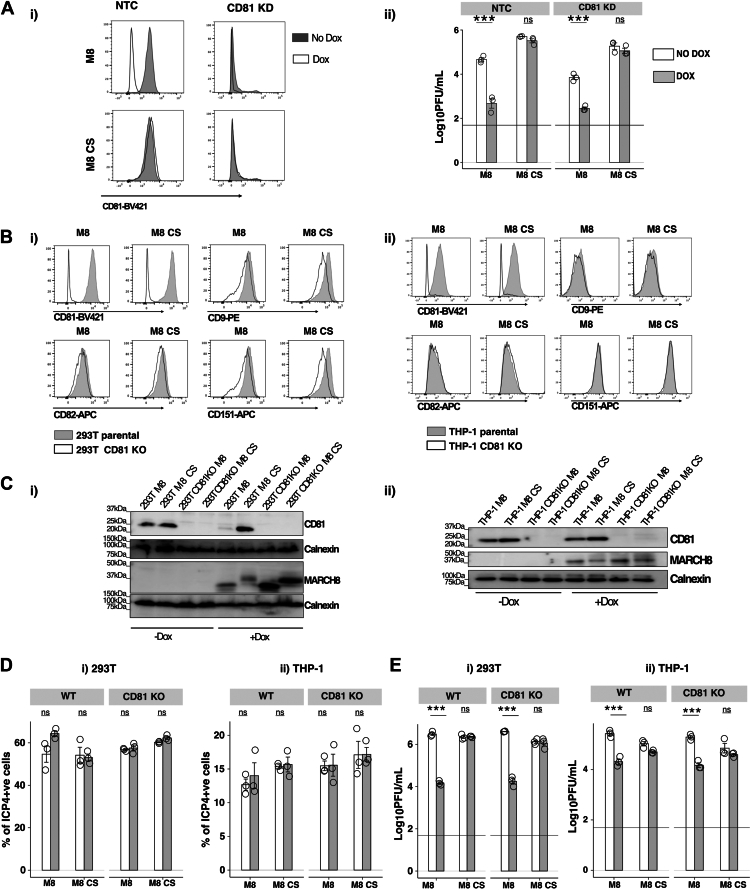
**Cellular CD81 does not contribute to M8-mediated restriction of HSV-1 replication.***A*, 293T cells with Dox-inducible expression of M8 or M8-CS were treated with CD81-specific or NTC siRNA and, 24 h later, incubated with (Dox) or without (No Dox) one ug/ml DOX for 24 h. Cells were then washed and (i) cell surface CD81 expression was determined by flow cytometry, or (ii) infected with HSV-1 (MOI 0.1) and virus titres in cell-free supernatants collected at 48 hpi were determined by plaque assay. Technical replicates from one of two independent experiments are shown. *B*, parental 293T and THP-1 cells with Dox-inducible expression of M8 or M8-CS were treated with CD81-specific guide RNA to generate CD81 KO cells. Flow cytometry was used to confirm KO of cell surface CD81 in (i) 293T or (ii) THP-1, with minimal effects on expression of the tetraspanins CD9, CD62, and CD151. *C*, parental or CD81 KO (i) 293T or (ii) THP-1 cells cultured for 24 h in the presence (+Dox) or absence (−Dox) of 1 μg/ml Dox were lysed and analysed by Western blot using a polyclonal Ab to detect CD81 or M8, or with a Calnexin loading control. *D* and *E*, parental or CD81 KO (i) 293T or (ii) THP-1 cells with Dox-inducible expression of M8 or M8-CS were cultured for 24 h in the presence or absence of 1 μg/ml Dox. *D*, cells were infected with HSV-1 KOS (MOI 4 and 10 for 293T and THP-1, respectively) and, at 5 hpi, cells were fixed, permeabilized and stained and stained with a mAb to the immediate early HSV-1 protein ICP4, followed by Alexa Fluor 488-conjugated chicken anti-mouse Ig. Technical replicates from one of two independent experiments are shown. *E*, cells were infected with HSV-1 KOS (MOI 0.1 and five for 293T and THP-1, respectively) and virus titres were determined in cell-free supernatants collected at 48 hpi. Technical replicates from one of 3 (293T) or one of 2 (THP-1) independent experiments are shown. The limit of detection is indicated as a *horizontal line*. Statistical analysis was performed using a mixed effects model utilizing data points from all experiments as described in Experimental procedures. ∗*p* < 0.01, ∗∗*p* < 0.001, ∗∗∗*p* < 0.0001 and ns = not significant.

Initial experiments used flow cytometry to confirm efficient siRNA-mediated knockdown of CD81 in M8 or M8-CS 293T cells (Fig. 6*A* (i)). In cells cultured without Dox, CD81 knockdown resulted in a modest reduction in HSV-1 titers relative to NTC-treated controls, indicating that CD81 is not essential for productive HSV-1 replication in 293T cells. Moreover, M8-mediated restriction of HSV-1 could proceed efficiently with or without CD81 knockdown (Fig. 6*A*(ii)). We also confirmed efficient siRNA-mediated knockdown of CD81 in THP-1 cells, however treatment of these cells with NTC siRNA rendered them refractory to supporting subsequent HSV-1 infection and growth (data not shown).

As an independent approach to assess the contribution of CD81 to M8-mediated restriction of HSV-1, we used CRISPR/Cas9 to generate 293T (M8 or M8-CS) and THP-1 (M8 or M8-CS) cells lacking expression of endogenous CD81. First, we used flow cytometry to confirm loss of cell-surface CD81 expression in 293T M8/M8-CS CD81 KO cells (Fig. 6*B*(i)) and in THP-1 M8/M8-CS CD81 KO cells (Fig. 6*B*(ii)), and that cell surface expression of related tetraspanins (CD9, CD82 and CD151) were unaffected in CD81. Moreover, Western blot confirmed effective loss of CD81 in 293T CD81 KO cells (Fig. 6*C*(i)) and in THP-1 CD81 KO cells (Fig. 6*C*(ii)), as well as Dox-inducible expression of M8 and M8-CS proteins in both cell types. Of note, levels of CD81 were reduced in 293T M8 and THP-1 M8 cells cultured in Dox, consistent with the ability of M8 to downregulate CD81 expression (8). Having confirmed the integrity of CD81 KO 293T and THP-1 cells, we next assessed early gene product expression (by ICP4 staining), as well as virus growth (by plaque assay), following infection with HSV-1. First, we did not observe any differences in the percentage of ICP4^+^ 293T ((Fig. 6*D*(i)) or THP-1 (Fig. 6*D*(ii)) cells, irrespective of whether M8 or M8-CS cells were cultured in the presence or absence of Dox. However, Dox-inducible M8, but not M8-CS, could effectively restrict HSV-1 growth in both 293T (Fig. 6*E*(i)) and in THP-1 cells (Fig. 6*E*(ii)), irrespective of endogenous CD81 expression. Together, these studies confirm that CD81 is dispensable for M8-mediated restriction of HSV-1 in both epithelial and macrophage cell lines.

### Cellular SAMHD1 is not essential for MARCHF8-mediated restriction of HSV-1 replication

**Figure 7:**
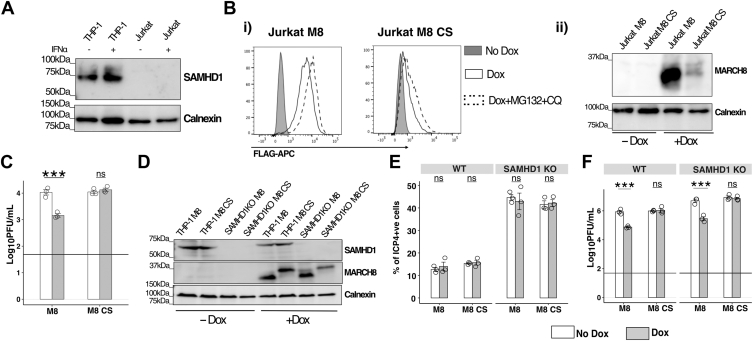
**Cellular SAMHD1 does not contribute to MARCHF8-mediated restriction of HSV-1 replication.***A*, THP-1 and Jurkat cells cultured 24 h in the presence (+) or absence (−) of 1000 mU/ml of recombinant human IFN-α were analyzed by Western blot with an anti-SAMHD1 mAb, and with a Calnexin loading control. *B*, Jurkat cells with Dox-inducible expression of FLAG-tagged M8 or M8-CS were cultured in No Dox, in Dox for 24 h or in Dox for 20 h before addition of MG132+CQ for the last 4 h. Dox-inducible protein expression was then determined (i) by flow cytometry using an anti-FLAG mAb to stain for intracellular protein expression, or (ii) by Western blot using a M8-specific polyclonal Ab, with a Calnexin loading control. *C*, Jurkat cells with Dox-inducible expression of M8 or M8-CS cultured for 24 h in the presence (Dox) or absence (No Dox) of 1 μg/ml Dox were then infected with HSV-1 KOS (MOI 0.1) and virus titres in cell-free supernatants collected at 48 hpi were determined by plaque assay. Technical replicates from one of three independent experiments are shown. *D–F*, PMA-differentiated parental (THP-1) and SAMHD1 knockout (SAMHD1 KO) THP-1 cells with inducible expression of M8 or M8-CS were cultured for 24 h in the presence (+Dox) or absence (-Dox) of 1 μg/ml Dox. *D*, cells were lysed and analysed by Western blot using a mAb to detect SAMHD1 or a polyclonal Ab to detect M8, or with a Calnexin loading control. *E*, cells were infected with HSV-1 KOS (MOI 10) and at 5 hpi, cells were fixed, permeabilized and stained and stained with a mAb to the immediate early HSV-1 protein ICP4, followed by Alexa Fluor 488-conjugated chicken anti-mouse Ig. Technical replicates from one of two independent experiments are shown. *F*, cells were infected with HSV-1 KOS (MOI 5), and virus titres in cell-free supernatant collected at 48 hpi were determined by plaque assay. Technical replicates from one of four independent experiments are shown. The *horizontal line* represents the limit of detection. Statistical analysis was done using a mixed-effects model utilizing data points from all experiments. ∗*p* < 0.01, ∗∗*p* < 0.001, ∗∗∗*p* < 0.0001 and ns = not significant.

SAMHD1 is a potent antiviral restriction factor against HIV-1 (37) and a number of herpesviruses, including HSV-1 (35, 38). Of interest, SAMHD1-mediated inhibition of HSV-1 in macrophages does not inhibit early gene expression but does block HSV-1 genomic replication and late gene expression (35). Moreover, modulation of CD81 has been shown to impact SAMHD1-mediated restriction of HIV-1 (39), and M8 is known to target and downregulate CD81 (8). Given established links between M8, CD81 and SAMHD1, we first utilized Jurkat cells, a human T cell line reported to lack expression of endogenous SAMHD1 (40). While SAMHD1 was readily detected in THP-1 cells by Western blot, it was not be detected in Jurkat cell lysates, even after stimulation with type I IFN (Fig. 7*A*). Next, we generated Jurkat cells with Dox-inducible expression of M8 or M8-CS (Fig. 7*B*) and confirmed that HSV-1 growth was effectively restricted by M8, but not M8-CS (Fig. 7*C*).

For THP-1 SAMHD1 KO cell lines, we used Western blot to confirm SAMHD1 KO, as well as expression of inducible M8 and M8-CS, in parental (WT) and in SAMHD1 KO THP-1 cells (Fig. 7*D*). Following infection with HSV-1, we observed no differences in the percentage of ICP4^+^ cells between WT and SAMHD1 KO cell lines cultured in the presence or absence of Dox (Fig. 7*D*(i)). When assessing virus growth in PMA-differentiated THP-1 cells, enhanced titers of virus were recovered from SAMHD1 KO compared to WT THP-1 cells cultured in No Dox conditions, consistent with the established role of SAMHD1 as an antiviral restriction factor for HSV-1 (35). However, similar levels of M8-mediated restriction of HSV-1 were observed following inducible expression of M8 in WT or SAMHD1 KO THP-1 cells (Fig. 7*F*). Together, these data confirm that SAMHD1 is an effective restriction factor against HSV-1 in differentiated THP-1 cells and that it is not essential for M8-mediated restriction of HSV-1 in these cells.

### Assessment of MARCHF8-mediated restriction of HSV-1 replication in the presence or absence of a functional cGAS-STING pathway

Double-stranded HSV-1 genomic DNA can be detected by the intracellular pattern recognition receptor (PPR) cGAS, resulting in downstream STING signaling and subsequent host immune responses (41). A recent study demonstrated that M8 can ubiquitinate cGAS, resulting in its downregulation and subsequent attenuation of the cGAS-STING pathway (15). Moreover, M8 KO mice showed enhanced antiviral responses and attenuated disease following intravenous HSV-1 infection (15). Considering these findings, we were interested to determine the net result of M8-mediated antiviral activity against HSV-1 *versus* its impact on downregulation of cGAS on productive HSV-1 replication.

**Figure 8:**
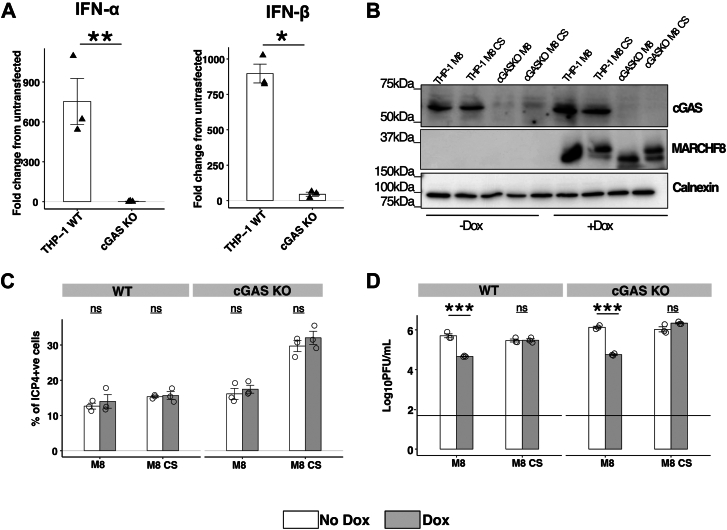
**MARCHF8-mediated restriction of HSV-1 in THP-1 cells proceeds in the presence of an intact cGAS signaling pathway.***A*, PMA-differentiated parental or cGAS KO THP-1 cells were transfected with 5 ug/ml HT-DNA for 8 h, then lysed for isolation of RNA. qPCR was then used to determine induction of IFN-α or IFN-β relative to untransfected cells. Technical replicates from one of two independent experiments are shown. *B*, parental or cGAS KO with Dox-inducible expression of M8 or M8-CS were generated. Cells cultured for 24 h in the presence (+Dox) or absence (−Dox) of 1 μg/ml Dox, were then lysed and analysed by Western blot using a rabbit mAb to detect cGAS or a rabbit polyclonal Ab to M8, or with a Calnexin loading control. *C* and *D*, parental or cGAS KO PMA-differentiated THP-1 cells with Dox-inducible expression of M8 or M8-CS were cultured for 24 h in the presence or absence of 1 μg/ml Dox. *C*, cells were infected with HSV-1 KOS (MOI 10) and, at 5 hpi, cells were fixed, permeabilized and stained with a mAb to the immediate early HSV-1 protein ICP4, followed by Alexa Fluor 488-conjugated chicken anti-mouse Ig. Technical replicates from one of two independent experiments are shown. *D*, cells were infected with HSV-1 (MOI 5) and virus titres in cell-free supernatants collected 48 hpi were determined by plaque assay. Technical replicates from one of three independent experiments are shown. Limit of detection is indicated by a *horizontal line*. Statistical analysis was performed using a mixed effects model utilizing data points from all experiments as described in Experimental procedures. ∗*p* < 0.01, ∗∗*p* < 0.001, ∗∗∗*p* < 0.0001 and ns = not significant.

To address this, we first transfected parental (WT) THP-1 and THP-1 cGAS KO cells with HT-DNA, a ligand known to stimulate the cGAS/STING signaling pathway (15). As seen in Figure 8*A*, HT-DNA transfection of parental, but not cGAS KO cells, resulted in induction of IFNα and IFNβ, consistent with expression of a functional cGAS-STING pathway in parental cells. Next, following transduction and selection of WT and cGAS KO THP-1 cells, we used Western blot to confirm expression of cGAS protein in WT, but not cGAS KO THP-1 cells, as well as expression of inducible M8 and M8-CS following culture in the presence of Dox (Fig. 8*B*). Following HSV-1 infection, no differences were detected in the percentage of ICP4^+^ cells detected in cells cultured in the presence or absence of Dox, irrespective of expression of M8 or M8-CS (Fig. 8*C*). In cells cultured in the No Dox condition, cGAS KO resulted in a modest increase in the titers of HSV-1 recovered from infected cells at 48 hpi (Fig. 8*D*). Following culture in the presence of Dox, inducible expression of M8, but not M8-CS, resulted in potent inhibition of HSV-1 growth, irrespective of whether THP-1 cells expressed endogenous cGAS or not (Fig. 8*D*). Moreover, across three independent experiments, M8-mediated restriction was more potent in cGAS KO cells but was still effective in reducing titres of HSV-1 in WT THP-1 cells. Thus, although M8 can attenuate the cGAS-STING pathway, inducible expression of M8 results in a net restriction of HSV-1 infectivity in differentiated THP-1 cells expressing a functional cGAS-STING pathway.

## Discussion

The E3 ligase MARCHF8 protein has been previously shown to mediate broad spectrum antiviral activity by targeting viral envelope glycoproteins from different RNA viruses, including HIV-1, VSV, IAV, CHIKV, RABV, and SARS-CoV-1 and -2 (17, 18, 22, 25, 20). In this study we demonstrate that the antiviral activity of MARCHF8 extends to human α-herpesviruses, namely HSV-1 and HSV-2. Of note, MARCHF8 did not inhibit replication of VACV, a member of the poxvirus family (28) and further studies are required to determine if MARCHF8 and/or other MARCH-family proteins can inhibit members of other DNA virus families. MARCHF8-mediated restriction of HSV-1 was associated with a block in viral genomic replication, as well as in subsequent steps in the viral replication cycle. Despite established links between MARCHF8, CD81, and SAMHD1, our studies demonstrated that the presence or absence of endogenous CD81 or SAMHD1 did not impact MARCHF8-mediated restriction of HSV-1. As such, the specific viral and/or cellular proteins targeted by MARCHF8 to inhibit HSV-1 replication are currently unknown.

For RNA viruses, the antiviral mechanism of action of MARCHF8 has been investigated in some detail. MARCHF8 has been shown to downregulate the HIV-1 Env and VSV-G envelope glycoproteins from the cell surface, thereby reducing their incorporation into nascent virions released from virus-producing cells (11, 18). Of interest, MARCHF8 targets virus envelope glycoproteins by at least two different mechanisms. Downregulation of VSV-G is dependent on the function of the N-terminal RING-CH domain of MARCHF8, which ubiquitinates residues in its lysine-rich CT, resulting in lysosomal degradation (22). While downregulation of HIV-1 Env also requires a functional RING-CH domain, a canonical tyrosine-based ^222^YxxL^225^ motif in the C-terminal CT of MARCHF8 is also required, and results in intracellular retention without degradation (22). MARCHF8 has also been reported to target lysine residues in the cytoplasmic tail of rabies virus (RABV) G protein, lymphocytic choriomeningitis virus (LCMV) GP protein, chikungunya virus (CHIKV) E2 proteins and the S glycoproteins of both SARS-CoV-1 and SARS-CoV-2 (20), although another study reported that MARCHF8-mediated downregulation of HIV-1 Env, EboV-GP and SARS-CoV-2 S glycoproteins was also observed using mutants lacking the CT of each viral glycoprotein (23). Irrespective of this, MARCHF8-mediated downregulation of RABV-G and SARS-CoV-1/2 S glycoproteins (20), as well as IAV infectivity (25), occurs *via* both ubiquitin-dependent and tyrosine motif-dependent pathways. Herein, we confirm that a functional RING-CH domain, as well as a tyrosine-based ^222^YxxL^225^ motif, are key requirements of MARCHF8 to mediate effective restriction of HSV-1 growth.

Our previous studies demonstrated that the two isoforms of MARCHF1 (M1.1 and M1.2) differed in their ability to restrict IAV replication (10). As for IAV, we have now demonstrated that M1.1, but not M1.2, also restricts HSV-1 replication. Moreover, our previous study demonstrated that a deletion mutant lacking the N-terminal cytoplasmic tail (N-CT) domain of M1.2 (M1_ΔN-CT) acquired the ability to recognize cellular substrates (*e.g.* CD44 and CD81), and to restrict IAV. Herein, we confirm that the M1_ΔN-CT mutant also acquired the ability to restrict HSV-1. Together, these findings suggest an inhibitory activity in the N-CT of M1.2 limits its ability to recognize certain substrates and to restrict IAV and HSV-1 replication.

For RNA viruses, MARCHF8 antiviral activity is generally associated with downregulation of viral envelope glycoproteins from the surface of virus producing cells, thereby reducing their incorporation into virions. However, in contrast to HIV-1 (11) and IAV (25), we demonstrate modest (293T) to no reduction (THP-1) in the infectivity per particle of virions released from HSV-1-infected cells in the presence of MARCHF8. While HSV-1 is an enveloped virus, the mechanisms driving virion assembly and egress are quite distinct to those of enveloped RNA viruses. Following HSV-1 genomic replication, packaging into preformed capsids and transport into the cytoplasm, the nucleocapsid and associated tegument proteins bud into the membrane of a cytoplasmic organelle, which contains all viral envelope glycoproteins to be expressed by the mature virion, in a process called secondary envelopment. From here, virions traffic to the periphery and are released by exocytosis (reviewed in (42)). Therefore, viral glycoproteins do not assemble at the cell surface prior to virus release, eliminating the opportunity for MARCHF8-mediated downregulation and reduced incorporation into budding virions. Consistent with this, we observed no major differences in infectivity per particle of virions released from HSV-1-infected cells in the presence or absence of MARCHF8.

Instead, our studies demonstrate that MARCHF8 restricts HSV-1 by interfering with replication and amplification of the viral genome. Antagonism at this level would therefore impact all subsequent steps in the HSV-1 replication cycle, including HSV-1 late gene expression, as well as viral assembly, egress and release. During lytic infection, HSV-1 genes are expressed through a temporal cascade of IE, E, and L viral genes transcribed chronologically by the cellular RNA polymerase II. The IE genes code for diverse viral proteins, including VP16 and ICP4, which can act as viral transcription factors to promote activation of E and L gene transcription (43), and E gene products include the viral replication machinery as well as additional viral proteins to facilitate HSV-1 genomic replication (43). L gene transcription commences following replication of the viral genome and generally code for viral structural proteins, including capsid proteins and glycoproteins. Given the complexity of HSV-1 transcription, many HSV-1 viral proteins could serve as potential targets for recognition by MARCHF8, resulting in ubiquitination and subsequent downregulation. Multiple cellular proteins are also enriched on viral replication forks and contribute to HSV-1 DNA replication (reviewed in (43)), and these could also represent potential targets for MARCHF8. Currently, it is not known which viral and/or host proteins are targeted by MARCHF8 to impair HSV-1 genomic replication, noting that its inhibitory effects are likely to be mediated in part through recognition and subsequent ubiquitination of exposed lysine residues within target proteins. However, as for HIV-1 Env (22), RABV-G and SARS-CoV-1/2 S glycoproteins (20), as well as IAV infectivity (25), tyrosine motif-dependent pathways are also critical for MARCHF8 restriction of HSV-1 infectivity.

In addition to cell-free spread of virions released from infected cells, all herpesviruses can spread ‘cell-to-cell’ whereby virions can pass directly through cell junctions to infect neighboring cells (44). Of interest, a recent study reported that MARCHF1 and MARCHF2 could inhibit pseudorabies virus (PRV), an α-herpesvirus that primarily infects pigs, and that restriction occurred at a post-entry stage *via* inhibition of viral glycoprotein-mediated cell-to-cell fusion (45). While we have not specifically addressed the impact of MARCHF8 on cell-to-cell spread of HSV-1, our findings that MARCHF8-mediated restriction of HSV-1 is associated with a block at an earlier stage in the virus replication cycle, specifically at the level of viral genomic replication, in indicative of a distinct and novel mechanism of antiviral activity.

In addition to restriction of multiple RNA viruses, our findings that MARCHF8 also inhibits human DNA viruses from the α-herpesvirus family adds a significant new dimension to our understanding of the breadth of its antiviral activities. Moreover, it paves the way for future studies to delve further into the spectrum of antiviral activity and the specific mechanism/s by which MARCHF8 mediates restriction of DNA viruses in general. Of interest, a recent study reported that MARCHF8 targets the cytoplasmic DNA sensor cGAS, resulting in its ubiquitination to dampen its DNA binding ability, subsequently impairing cGAMP production and suppressing cGAS-mediated antiviral signaling (15). While the cGAS-STING pathway is defective in most cancerous cell lines (46), our studies using THP-1 cells confirm that MARCHF8 can restrict HSV-1 replication even in cells expressing a functional cGAS-STING pathway. This ability of MARCHF8 to restrict HSV-1 genomic replication in the face of attenuated cGAS-mediated antiviral signaling places it with other antiviral restriction factors such as ADAR1, SAMHD1, and MORC3, which limit IFN-inducing PRRs or IFN-signaling yet also exert direct antiviral activity (47, 48, 49), with MORC3 also demonstrating direct antiviral activity against HSV-1 (50). Recent research indicates that this may be a broader strategy by which the immune system controls both viral spread and inflammation in the host, as well as self-guarding antiviral factors. Moreover, HSV-1 activates multiple PRRs system of the innate immune system other than cGAS, including MDA5, TLR3, and TLR9 (51), which all act at different stages of viral infection and replication. Thus, our study places MARCHF8 within a larger, orchestrated response to control HSV-1, which acts to protect the host from damage both through infection and inflammation.

## Experimental procedures

### Cell lines

Human monocytic THP-1 (ATCC TIB-202) cells and Madin-Darby Canine kidney (MDCK) cells (ATCC CCL-34) were maintained and passaged in RPMI 1640 medium supplemented with 10% (v/v) foetal calf serum (FCS, Gibco, Thermo Fisher Scientific), 2 mM L-glutamine (Gibco), non-essential amino acids (Gibco), 0.55% (v/v) sodium bicarbonate (Gibco), 20 mM HEPES (Gibco), 200 Units (U)/ml penicillin (Gibco), and 200 μg/ml streptomycin (Gibco). For all experiments, THP-1 were seeded and differentiated in 25 ng/ml of phorbol myristate acetate (PMA) in culture media. BEAS-2B cells were maintained in Ham's F-12K (Kaighn's) Medium (Gibco) supplemented with 10% (v/v) FCS, and supplements as above. Human embryonic kidney (HEK) 293T (ATCC CRL-3216), HeLa (ATCC CCL-2), human epithelial type 2 (HEp-2), and African Green monkey kidney Vero cells (CSL, Parkville Australia) were maintained and passaged in Dulbecco's Modified Eagle Medium (DMEM) (Gibco) supplemented with 10% (v/v) FBS and supplements as above. All cell lines were cultured at 37 °C in a humidified incubator with 5% (v/v) CO2.

### Virus

Herpes simplex virus (HSV)-1 KOS, SC-16, and F strains and HSV-2 strain 186, as well as a recombinant HSV-1 KOS virus with green fluorescent protein (GFP) inserted to disrupt the TK locus and expressed under the control of a HCMV promoter (30) were all kindly provided by Prof. Francis Carbone, The University of Melbourne, Australia. Recombinant HSV-1 viruses on the RE strain background expressing EGFP and/or red fluorescent protein (RFP) under the control of the HSV-1 gB or gC promoters, respectively (34), referred to as double fluorescent (DF) HSV-1, were a kind gift from Paul (Kip) Kinchington, University of Pittsburgh. Viruses were propagated in Vero cells, and titers of infectious virus were determined by standard plaque assay on Vero cells. Vaccinia virus (VACV) expressing full length ovalbumin from the p7.5 (early and late) promoter placed into the (J2R) TK region of VACV WR, such that TK is non-functional (52) (VACV-OVA), was a kind gift from Prof. David Tscharke, Australian National University, Australia.

### Generation of cell lines with doxycycline-inducible protein expression

To produce cell lines with doxycycline (Dox)-inducible expression of MARCHF1 or MARCHF8, a three-step cloning strategy was used as described (10, 25). Briefly, codon-optimized MARCHF1 isoform 1 (CCDS54814.1), MARCHF1 isoform 2 (CCDS3806.1) or MARCHF8 (CCDS7213.1), each with a N-terminal FLAG-tag, were synthesized as ‘geneblocks’ (GeneArt Strings DNA Fragments; Invitrogen). The coding sequence was then sequentially cloned into the pTRE-Tight plasmid vector containing a tight TRE promoter that confers Dox inducibility. Fragments were then subcloned into the pFUV1-mCherry lentivirus transfer plasmid (kindly provided by Professor Marco Herold, The Walter and Eliza Hall Institute, Australia) (53). A control cell line (CTRL) expressing cytoplasmic hen egg ovalbumin lacking the sequence for cell surface trafficking was also prepared. Lentivirus stocks were produced by co-transfecting packaging plasmids pMDL (gag and pol) (0.25 μg) and pRSV-REV (0.12 μg), as well as pMD2G.VSVg envelope plasmid (0.15 μg), and pFUV1-mCherry-MARCHF1/8 transfer plasmid (0.49 μg) into 293T cells using Lipofectamine 2000 (Invitrogen). At 48 h post-transfection the media was harvested and used to transduce either 293T, THP-1, BEAS-2B or HeLa cells in media supplemented with 1ug/ml polybrene and mCherry-positive cells were sorted 72 h later using a BD FACS Aria III Cell Sorter (BD BioScience) and expanded for use in experiments. The MARCHF1.2 isoform was used in all experiments unless otherwise stated.

### Generation of MARCHF8 and MARCHF1 mutants, as well as chimeric MARCHF1 and MARCHF8

MARCHF8 (M8) mutants and chimeric MARCHF1 (M1) or M8 proteins were generated as previously described (10, 25). M8 mutants included (i) a M8 CS mutant (54), where all cysteine (C) residues at positions 80, 83, 97, 99, 110, 123, and 126 were replaced with serine (S) residues, (ii) M8 W114A (55) where a tryptophan (W) at position 114 was replaced by alanine (A), and (iii) two tyrosine motif mutants M8-^222^Axx^L225^ and M8-^232^AxxV^235^ (22). Additional mutants included N-CT domain mutants, where the N-CT domains of M1 and M8 were identified based on the position of their respective RING-CH domains, and included (i) M8 with N-CT deletion (M8_ΔN-CT), M1 with N-CT deletion (M1_ΔN-CT), and M1 with sequential removal of specific lysine (K) residues (which might serve as potential targets for autoubiquitination) from the N-CT to generate M1 N-CT-Δ15, N-CT-Δ16, N-CT Δ29 and N-CT-Δ40 mutants. In addition, we generated a chimeric M8 expressing the N-CT of M1 (M8_M1 N-CT) and M1 expressing the N-CT of M8 (M1_M8 N-CT). Previous studies from our laboratory describe the generation of 293T cell lines with Dox-inducible expression of mutant and chimeric M1/M8 proteins, as well as their characterization by flow cytometry for intracellular FLAG (as a measure of protein expression) and CD86 (using downregulation to confirm functional protein expression) for M1, M8, M8 CS, M8 114A, M8-^222^Axx^L225^, M8-^232^AxxV^235^, M8_ΔN-CT, and M1_ΔN-CT (25) and for M1 N-CT-Δ15, N-CT-Δ16, N-CT D29, and N-CT-Δ40 mutants (10).

### Flow cytometry for detection of cell-surface proteins or intracellular FLAG expression

293T, THP-1, BEAS-2B or HeLa cells with Dox-inducible protein expression were seeded and cultured overnight at 37°C. To induce M1/M8 protein expression, cells were treated with 1 μg/ml of Dox (Sigma-Aldrich) for 24 h at 37 °C and, at 20 h post-Dox induction, 10 μM of the proteasomal inhibitor MG132 (Sigma-Aldrich) and 20 μM of the lysosomal inhibitor chloroquine (CQ) was added to inhibit intracellular protein degradation. At 24 h after addition of Dox cells were detached and stained with fixable viability dye eFluor 450 (eBioscience), and with combinations of phycoerythrin (PE)-conjugated anti-CD9 (Clone B19a, BioLegend), Brilliant Violet (BV)421-conjugated anti-CD81 (Clone JSS-81, BD Bioscience), Allophycocyanin (APC)-conjugated anti-CD82 (Clone ASL-24, BioLegend), BV711-conjugated anti-CD86 (Clone FUN-1, BD BioScience), and/or APC-conjugated anti-CD151 (Clone 50–6, Biolegend)), washed and fixed with 4% (v/v) paraformaldehyde in PBS and then analyzed by flow cytometry. For intracellular staining, cells were fixed with 4% (v/v) paraformaldehyde in PBS, permeabilized with 0.5% (v/v) Triton X-100 in PBS, stained with anti-FLAG-APC mAb (Clone L5, Biolegend) and washed and analyzed by flow cytometry. Cells were acquired using a LSR Fortessa Flow Cytometer (BD Bioscience) and analyzed using FlowJo analysis software version 10.9.0.

To test impact of HSV-1 infection on expression of FLAG-tagged M1/M8, cells with DOX-inducible expression of M1/M8 (293T, BEAS-2B or HeLa cells) were seeded, cultured overnight and then cultured in the presence (DOX) or absence (No DOX) of 1 μg/ml of Doxycycline (Sigma-Aldrich) for 24 h at 37°C. Cells were then mock-infected or infected with HSV-1-TK-GFP (MOI 2) for 8 h. In some experiments cells were infected with HSV-1-TK-GFP for 6 h prior to addition of MG132 and chloroquine for 2 h. At 8 hpi, cells were detached, stained with viability dye (eFlour 450), fixed with 4% (v/v) paraformaldehyde in PBS and then permeabilized in 0.5% (v/v) Triton X-100 in PBS and stained with an anti-FLAG mAb conjugated to APC (Clone L5, Biolegend). Cells were then washed and acquired using a LSR Fortessa Flow Cytometer (BD Bioscience) and analyzed using FlowJo analysis software version 10.9.0.

### Western blot

Cells with Dox-inducible protein expression were cultured overnight at 37 °C, then treated with 1 μg/ml Dox for 24 h at 37 °C and, at 20 h post-Dox induction, 10 μM of MG132 and 20 μM of CQ were added to inhibit intracellular protein degradation. Cells were then lysed in in RIPA lysis buffer (1%(v/v) Triton-X-100, 150 mM NaCl, 1% (w/v) Na deoxycholate, 0.1% SDS (v/v), and 50 mM Tris (pH 7.5)) supplemented with protease inhibitors (complete Mini Protease Inhibitor Cocktail; Roche, Basel, Switzerland). Cell lysates were incubated with 5x SDS-sample buffer (50 mM Tris, 0.05% (w/v) bromophenol blue, 2% (v/v) sodium dodecyl sulfate (SDS), with or without 20 mM dithiothreitol (DTT)) at 95 °C for 15 min and then separated by gel electrophoresis using 10% or 15% (v/v) SDS-polyacrylamide gels. Samples were subsequently transferred to a PVDF membrane and blocked in blocking buffer (5% (w/v) bovine serum albumin, 0.1% Tween-20 (Sigma-Aldrich) in PBS) for 1 h at room temperature (RT). Membranes were then incubated with either anti-FLAG rat mAb (Clone L5, BioLegend), anti-MARCHF8 rabbit polyclonal (p)Ab (Proteintech), anti-CD81 mouse mAb (Clone JS-81, Biosciences), anti-SAMHD1 mouse mAb (Clone OTI3F5, ThermoFisher Scientific), anti-cGAS rabbit mAb (Clone D1D3G, Cell Signalling), or with anti-calnexin rabbit pAb (Abcam) overnight at 4 °C. After washing with PBS containing 0.05% (v/v) Tween-20, membranes were probed with appropriate secondary antibodies, namely rabbit anti-mouse IgG horseradish peroxidase (HRP, DAKO), donkey anti-rabbit IgG HRP (Abcam), donkey anti-rat HRP (DAKO) or anti-rabbit IgG AlexaFluor 647 (Invitrogen), for 2 h at RT. After washing, bound antibodies were detected after scanning for fluorescence or chemiluminescence using Amersham ImagerQuant 800 and analyzed using Fiji ImageJ software.

### Virus infection assays

Cells seeded into 24-well tissue culture plates were cultured overnight at 37°C and cells with Dox-inducible protein expression were then cultured in the presence or absence of 1 μg/ml of Dox for a further 24 h. For infection, cells were washed and then incubated for 1 h at 37 °C with virus diluted to the appropriate multiplicity of infection (MOI, as indicated) in serum-free media. After washing, cells were cultured in serum-free media with (Dox) or without (No Dox) 1 μg/ml of Dox as indicated. At 2, 24 or 48 h post-infection (hpi) cell supernatants were collected, clarified by centrifugation and titres of infectious virus were then determined by plaque assay on Vero cells with titers expressed as plaque-forming units (PFU) per mL (56).

### Flow cytometry for detection of HSV-1-infected cells

HSV-1-infected cells were detached, washed, transferred to U-bottom 96-well plates (Corning) and then stained with fixable viability dye eFluor 780 (eBioscience). Cells were then fixed in 2 to 4% (vol/vol) PFA, permeabilized with 0.5% (vol/vol) Triton X-100 in PBS for 10 min at RT, and then washed in permeabilization buffer (PBS containing 0.25% [vol/vol] Triton X-100, 1% [vol/vol] FCS, and 1 mM EDTA) and stained with a mouse mAb specific for HSV-1 ICP5 (VP5) (Abcam), followed by chicken anti-mouse IgG conjugated to Alexa Fluor 488 (Invitrogen). Cells infected with recombinant HSV-1 expressing GFP and/or RFP were stained with fixable viability dye eFluor 780 (eBioscience), fixed in 2% (vol/vol) PFA and then washed and analyzed by flow cytometry. Samples were acquired using a LSR Fortessa Flow Cytometer (BD Bioscience) and analyzed using FlowJo analysis software version 10.9.0.

### siRNA knockdown of endogenous CD81 or MARCHF8

Cells were seeded at 24-well tissue culture plates, cultured overnight at 37°C and then media was replaced by OPTIMEM (Gibco) supplemented with 5% FCS. For CD81 knockdown, CD81 or non-targeting control (NTC) siRNA (M-017257-02-0005 siGENOME Human CD81 siRNA SMARTpool (targeting four different regions) and DHA-D-001910-10-05 Accell Non-targeting, Dharmacon - Horizon Discovery) were mixed with serum-free OPTIMEM and Lipofectamine RNAiMAX Transfection Reagent (Thermo Fisher) for 5 min at RT and 50 nM of siRNA/LipoRNAiMAX was then added to cells and incubated at 37 °C for 48 h. After washing, cells were either (i) stained for cell-surface of CD81 and assessed by flow cytometry to determine knockdown efficiency, or (ii) infected with the HSV-1 strain KOS at the appropriate MOI (as indicated) and virus titres in clarified supernatants were determined at various times post-infection. M8 knockdown was performed essentially as described (25), with A549 cells incubated with 10 nM M8 or NTC siRNA (DHA-E-HUMAN-XX-0005 Accell SMARTpool (targeting four different regions), Dharmacon - Horizon Discovery) for 48 h prior to (i) qPCR for M8 mRNA expression as described (25), (ii) infection with DF HSV-1 virus (MOI 1) for 18 h before the percentage of GFP- and RFP-positive cells were determined by flow cytometry or (iii) infection with HSV-1 KO virus (MOI 0.001) for 36 h before virus titers in clarified supernatants were determined by plaque assay.

### CRISPR/Cas9 knockout cells deficient in CD81, SAMHD1 or cGAS

CD81 knockout (KO) 293T and THP-1 cells were generated *via* the CRISPR/Cas9 endonuclease system using SF Cell Line 4D-Nucleofector X Kit (Lonza). Briefly, cells were seeded, cultured for 24 h, and then resuspended in 104 pmol Alt-R Cas9 as well as 180 pmol of two gRNAs targeting CD81 (sgRNA1: 5′ *GAUCACGCCUCCAGCCAGCU* 3′, sgRNA2: 5′ *CUGGCUGGAGGCGUGAUCCU* 3′, Synthego). Cells were then electroporated in a Nucleocuvette Strip (Lonza), expanded in culture and cells lacking cell-surface CD81 expression were selected and sorted using a BD FACS Aria III Cell Sorter (BD BioScience). Parental and SAMHD1 knockout THP-1 cells (a kind gift from Professor Thomas Gramberg, University of Erlangen-Nuremberg, Germany) were cultured in RPMI 1640 medium containing supplements (as above), as well as 100 μg/L G418 (Gibco) and 2.5 ug/ml puromycin (Gibco). cGAS KO THP-1 (a kind gift from Prof. Si Ming Man, The Australian National University) were cultured in RPMI 1640 medium containing supplements.

### Quantitation of HSV-1 genomic DNA by qPCR

Levels of HSV-1 genomic DNA in virus-infected cells or in clarified supernatants from infected cells were determined as described (57). Briefly, total cellular DNA was extracted using a DNeasy Blood & Tissue Kit (Qiagen) and TaqMan-based real-time PCR was performed using 100 ng genomic DNA, along with primers (forward 5′-TTGTCTCCTTCCGTGTTTCAGTTAGCC-3′; reverse 5′-GGCTCCATACCGACGATCTGCG-3′), and probe specific for the HSV-1 TK gene (5′-6-carboxyfluorescein[FAM]-CCATCTCCCGGGCAAACGTGC-MGBNFQ-3′, ThermoFisher) and TaqMan Fast Advanced Master Mix (ThermoFisher) under appropriate cycling conditions (50 °C for 2 min and 95 °C for 10 min, 40 cycles of 95 °C for 15 sec and 60 °C for 1 min). A 10-point standard curve was generated using serial 10-fold dilutions of pGEMT-Easy TK qPCR plasmid (a kind gift from David Tscharke, Australian National University) and the copy number of HSV-1 DNA from each sample was calculated relative to the standard curve and results were expressed as log_10_DNA copies/ml. The limit of detection (LOD) for the assay was set to 2. The ratio between the titer of infectious HSV-1 (plaque assay) and the total number of HSV-1 particles released (qPCR) was then used to determine the infectivity per particle as described (58).

### Detection of endogenous MARCHF1 or 8 mRNA expression by qPCR

Detection of endogenous MARCHF1 and eight mRNA was performed as described previously (10, 25). Briefly, total RNA was extracted using a RNeasy mini kit (Qiagen) and was converted to cDNA using a SensiFAST cDNA synthesis kit (Bioline). A SYBR green-based qPCR assay was then used to determine expression of *MARCHF1/8* and housekeeping genes *GAPDH* (glyceraldehyde 3-phosphate dehydrogenase) and TBP (TATA-binding protein) using a SensiFAST SYBR Lo-ROX kit. The specific primers used were as follows: MARCHF8, forward (5′-AGCCACTGAGAAAATGGGAGAAG-3′) and reverse (5′-TGTCACTGAGCACATGATCTTCC-3′); MARCH 1.1, forward (*5′-CGCCTCACAAACCTCCACAT-3′)* and reverse (*5′-TGTTGGGCTGCTTGCTTTTG-3′);* MARCH 1.2, forward (*5′-ATGACCAGCAGCCACGTTT-3′*) and reverse (*5′-CAAGTTAGATAATTTGGCATCTTGG-3′); GAPDH*, forward (5′-*TGAAGGTCGGAGTCAACGG*-3′) and reverse (5′-G*GCAACAATATCCACTTTACCAGAG*-3′); and *TBP*, forward (5′-*GCACTGATTTTCAGTTCTGG*-3′) and reverse (5′-*GCTGGAAAACCCAACTTCTGT*-3′). Data acquisition was performed using the QuantStudio 7 Pro (Applied Biosystems), and MARCHF8 expression relative to housekeeping genes was determined using the 2^-ΔCT^ method, while expression of MARCHF1/8 was determined relative to mock-treated cells using the 2^-ΔΔCT^ method (25).

### Detection of IFNa and IFNb mRNA by qPCR to confirm functionality of the cGAS/STING pathway

Parental and cGAS KO THP-1 cells seeded and cultured overnight at 37°C were transfected with 5 μg/ml HT-DNA (Merck) using Lipofectamine 2000 (Invitrogen) in OPTIMEM (Gibco). After 8 h, total RNA was extracted using a RNeasy mini kit (Qiagen, Germany) and was converted to cDNA using a SensiFAST cDNA synthesis kit (Bioline). qPCR (QuantStudio 7 pro) was performed to determine expression of IFNα1 (forward 5′-*GACTCCATCTTGGCTGTGA*-3′ and reverse 5′- *TGATTTCTGCTCTGACAACCT*-3′) or IFNβ (forward 5′-*CAGTCCTGGAAGAAAAACTGGAGA*-3′ and reverse 5′-*TTGGCCTTCAGGTAATGCAGAA*-3′) or *GAPDH*, (forward 5′-*TGAAGGTCGGAGTCAACGG*-3′ and reverse 5′-G*GCAACAATATCCACTTTACCAGAG*-3′) Relative expression of IFNα and IFNβ was determined after normalizing to housekeeping gene and then relative to untransfected cells using the 2^-ΔΔCt^ method (59).

### Statistical analysis and data visualization

Graphs and statistical analysis (as indicated in the figure legends) was generated using R-Studio. Statistical analysis was performed by utilizing a mixed effects model (*nlme* package in R) to estimate the fixed effects (*e.g.* difference between Dox vs No Dox or siRNA vs NTC while controlling for similarities between technical replicates (random effects) in each independent experiment (biological replicates)). This model utilizes the Satterthwaite approximation for calculating *p*-values (*via* the *LmerTes*t package). To ensure model assumptions were met, we inspected the normality of the residuals using QQplots and used the Shapiro–Wilk test.

The mixed-effect model was used as it allows for a more accurate estimate of the variation associated which each sample after accounting for technical replication. For clarity of visualization representative data from one experiment (technical replicates) is shown in figures, but significance was calculated utilizing all data points from each independent (biological) experiments (*i.e.* if three independent experiments were performed, each with three technical replicates, nine data points were used in the model).

## Data availability

The majority of data are contained within the manuscript and any additional data can be shared upon request (Prof. Patrick Reading: preading@unimelb.edu.au)

## Supporting information

This article contains supporting information (10).

## Conflict of interest

The authors declare that they have no conflicts of interest with the contents of this article.
