# Supplementary material for: The E3 ubiquitin ligase MARCHF8 restricts HSV-1 infection by inhibiting replication of the viral genome
Source: J Biol Chem. 2025 Aug 6;301(9):110567. doi: 10.1016/j.jbc.2025.110567 (PMC12446638; doi:10.1016/j.jbc.2025.110567)
Supplement: Supplementary_Figs [file mmc1.docx]

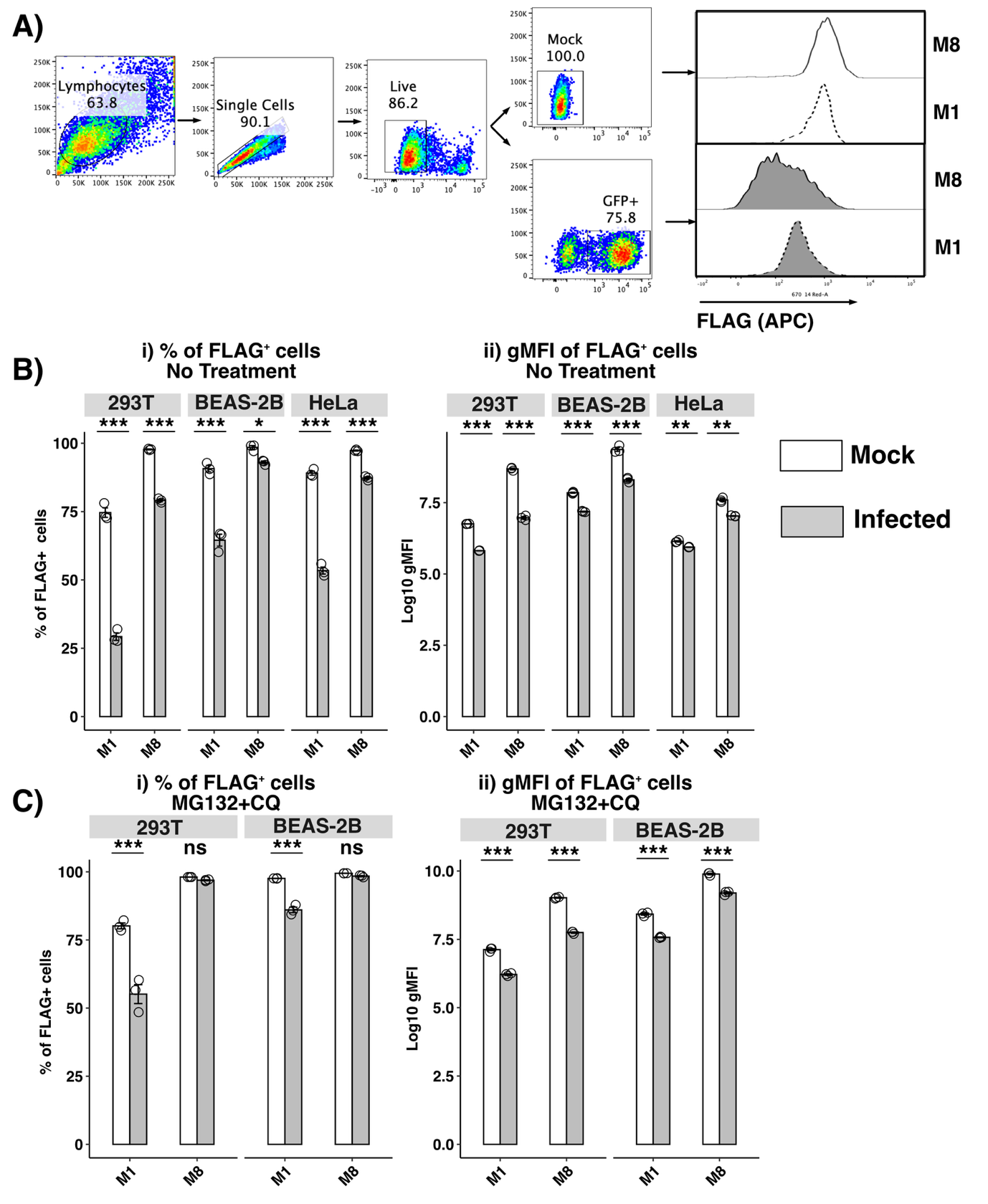


**Figure S1: Impact of HSV-1 infection on expression of DOX-inducible M1/M8 proteins in different cell types. A)** 293T cells with doxycycline (Dox)-inducible expression of FLAG-tagged MARCHF1 (M1) or MARCHF8 (M8) were cultured in 1ug/mL Dox for 24 hr and then mock infected or infected with HSV-1 KOS-TK-GFP (MOI 2). At 8 hpi cells were fixed, live/dead stained and then stained for intracellular expression of FLAG-tagged M1/M8 proteins and analysed by flow cytometry. Dot plots to show gating strategy for identification of FLAG^+^ cells in mock- vs HSV-1-infected cells. Representative histograms show expression of FLAG-tagged M1/M8 in mock vs HSV-1-infected cells (white and grey histograms, respectively). **B)** 293T, BEAS2b and HeLa with Dox-inducible expression of FLAG-tagged M1 or M8 were cultured in 1ug/mL Dox for 24 hr and then mock-infected or infected with HSV-1 KOS-TK-GFP (MOI 2). At 8 hpi cells were fixed, live/dead stained and then stained for intracellular expression of FLAG-tagged M1/M8 proteins and analysed by flow cytometry. Data shown represent **(i)** percentage of FLAG^+^ cells, and **(ii)** Log_10_ gMFI of FLAG^+^ cells in mock vs GFP^+^ (HSV-1-infected) cells. Technical replicates from 1 of 3 independent experiments are shown. **C)** 293T and BEAS2b with Dox-inducible expression of FLAG-tagged M1 or M8 were cultured in 1ug/mL Dox for 24 hr and then mock-infected or infected with HSV-1 KOS-TK-GFP (MOI 2) and cultured for 6 hrs before addition of MG132 and chloroquine (MG132+CQ) for the last 2 hr. At 8 hpi cells were fixed, live/dead stained and then stained for intracellular expression of FLAG-tagged M1/M8 proteins and analysed by flow cytometry. Data shown represent **(i)** percentage of FLAG^+^ cells, and **(ii)** Log_10_ gMFI of FLAG^+^ cells in mock vs GFP^+^ (HSV-1-infected) cells. Technical replicates from 1 of 2 independent experiments are shown. Statistical analysis was performed using a mixed effects model utilizing data points from all experiments, as described in Materials and Methods. * *P*<0.01, ** *P*<0.001, *** *P*<0.0001 and ns = not significant.


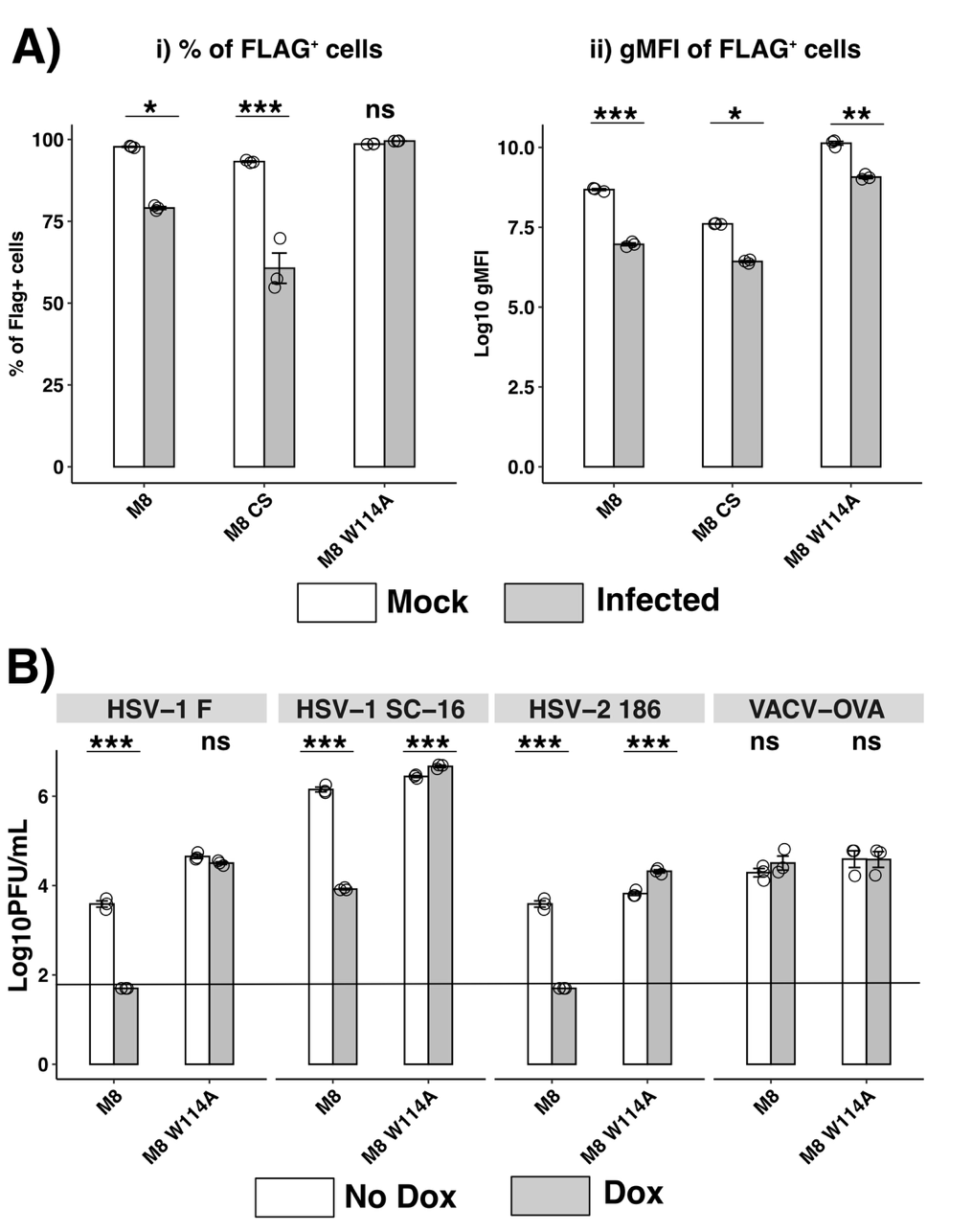


**Figure S2: A functional E3 ligase domain is required for antiviral activity against α-herpesviruses. A)** 293T cells with Dox-inducible expression of FLAG-tagged M8 or M8 E3 ligase mutants (M8 CS or M8 W114A) were cultured in the presence of 1ug/mL Dox for 24 hr and then mock infected or infected with HSV-1 KOS-TK-GFP (MOI 2). At 8hpi cells were fixed, live/dead stained and then stained for intracellular expression of FLAG-tagged proteins and analysed by flow cytometry. Data shown represent **(i)** percentage of FLAG^+^ cells, and **(ii)** Log_10_ gMFI of FLAG^+^ cells in mock vs GFP^+^ (HSV-1-infected) cells. Technical replicates from 1 of 2 independent experiments are shown. **B)** 293T cells with Dox-inducible protein expression were cultured for 24 hr in the presence (Dox) or absence (No Dox) of Dox and then infected with HSV-1 (strains F or SC-16), HSV-2 (strain 186) or VACV-OVA (all at MOI 0.1). Virus titres in clarified supernatants collected at 48 hpi were determined by plaque assay. Technical replicates from 1 of 2 independent experiments are shown. Limit of detection for plaque assay results are shown as a horizontal line. Statistical analysis was performed using a mixed effects model utilizing data points from all experiments, as described in Materials and Methods. * *P*<0.01, ** *P*<0.001, *** *P*<0.0001 and ns= not significant.


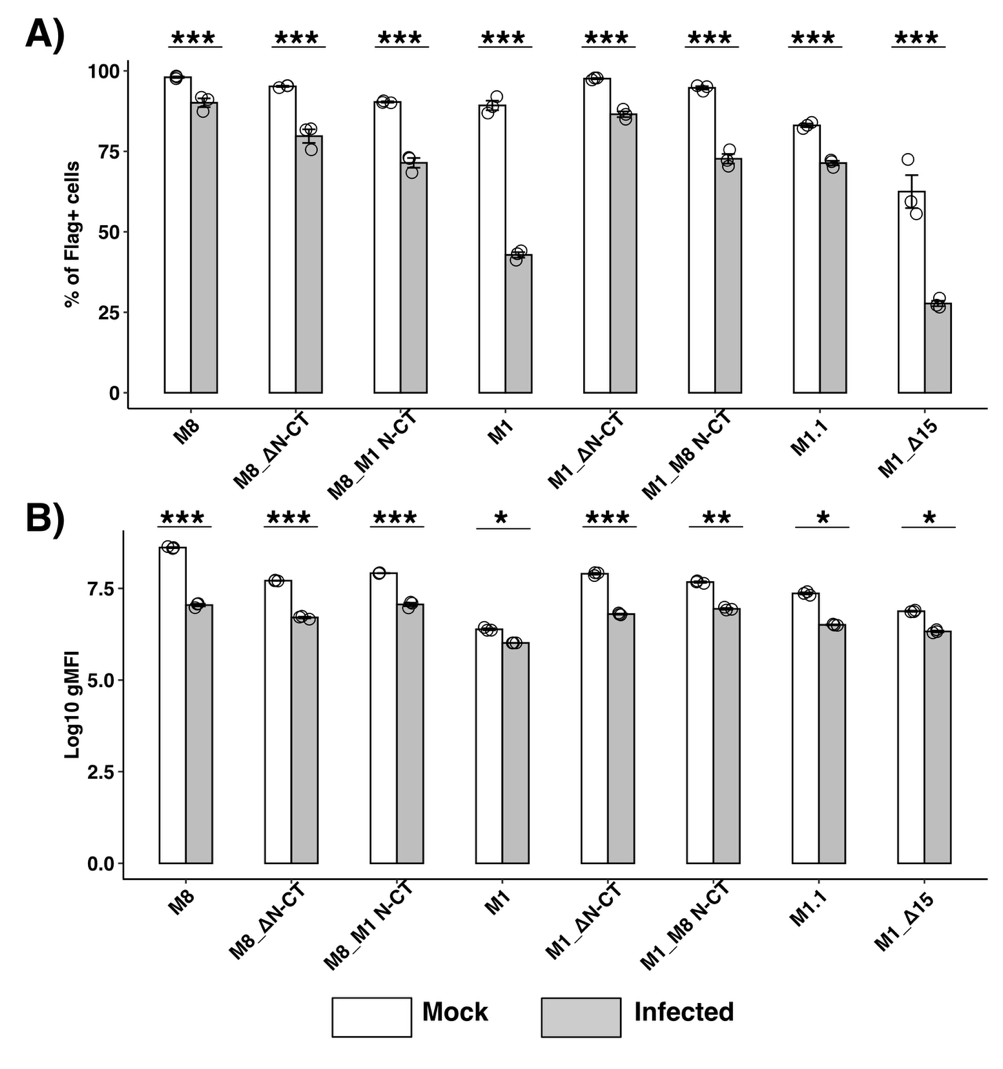


**Fig S3: Impact of HSV-1 infection on expression of DOX-inducible M1/M8 deletion mutants and chimeric proteins in 293T cells.** 293T cell lines with DOX-inducible expression of (i) parental M8, M1 or M1 isoform 1 (M1.1), or (ii) M8 with N-CT deletion (M8_ΔN-CT) or substitution for the N-CT of M1 (M8_M1 N-CT), or M1 with N-CT deletion (M1_ΔN-CT) or substitution for the N-CT of M8 (M1_M8 N-CT) were generated and characterized as described [1]. Cells were cultured in the presence of 1ug/mL Dox for 24 hr and then mock infected or infected with HSV-1 KOS-TK-GFP (MOI 2). At 8hpi cells were fixed, live/dead stained and then stained for intracellular expression of FLAG-tagged proteins and analysed by flow cytometry. Data shown represent **(i)** percentage of FLAG^+^ cells, and **(ii)** Log_10_ gMFI of FLAG^+^ cells in mock vs GFP^+^ (HSV-1-infected) cells. Technical replicates from 1 of 2 independent experiments are shown. Statistical analysis was performed using a mixed effects model utilizing data points from all experiments, as described in Materials and Methods. * *P*<0.01, ** *P*<0.001, *** *P*<0.0001 and ns= not significant.


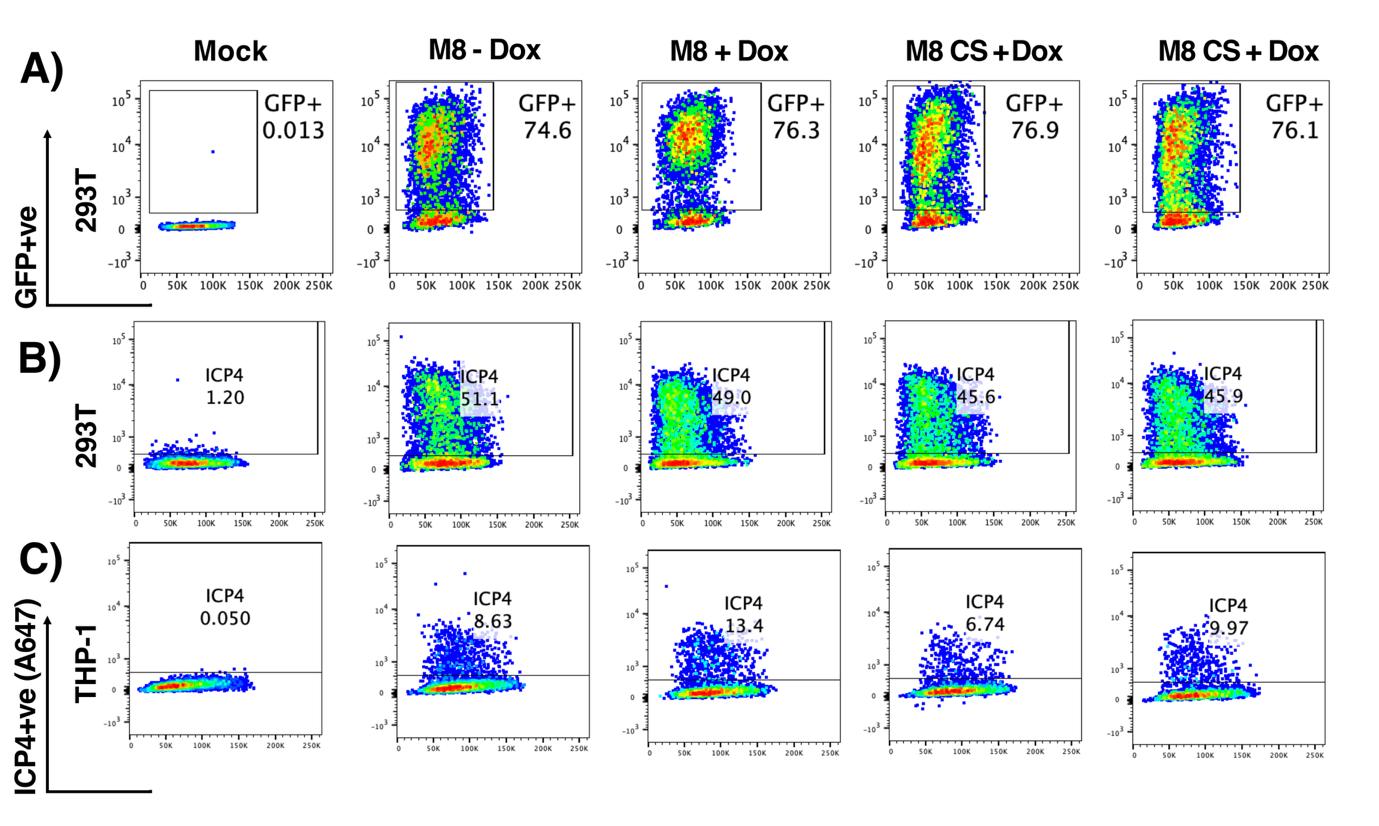


**Figure S4: MARCHF8 does not inhibit HSV-1 entry and translocation to the nucleus in 293T or THP-1 cells.** 293T cells with Dox-inducible expression of FLAG-tagged M8 or M8-CS were cultured in the presence (+Dox) or absence (-Dox) of 1ug/mL Dox for 24 hr and then infected with **A)** HSV-1 KOS-TK-GFP (MOI 2), or **B)** HSV-1 KOS (MOI 4) and analysed at 8 hpi. **A)** Cells were fixed and the percentage of GFP^+^ cells was determined by flow cytometry. Representative dot plots are shown. **B)** Cells were fixed, permeabilized and stained with a mAb to the immediate early HSV-1 protein ICP4, followed by Alexa Fluor 488-conjugated chicken anti-mouse Ig. Representative dot plots are shown. **C)** THP-1 cells with Dox-inducible expression of FLAG-tagged M8 or M8-CS were cultured in the presence (Dox) or absence (No Dox) of 1ug/mL Dox for 24 hr and then infected with HSV-1 KOS (MOI 10) and analysed at 5 hpi. Cells were fixed, permeabilized and stained with a mAb to the immediate early HSV-1 protein ICP4, followed by Alexa Fluor 488-conjugated chicken anti-mouse Ig. Representative dot plots are shown.


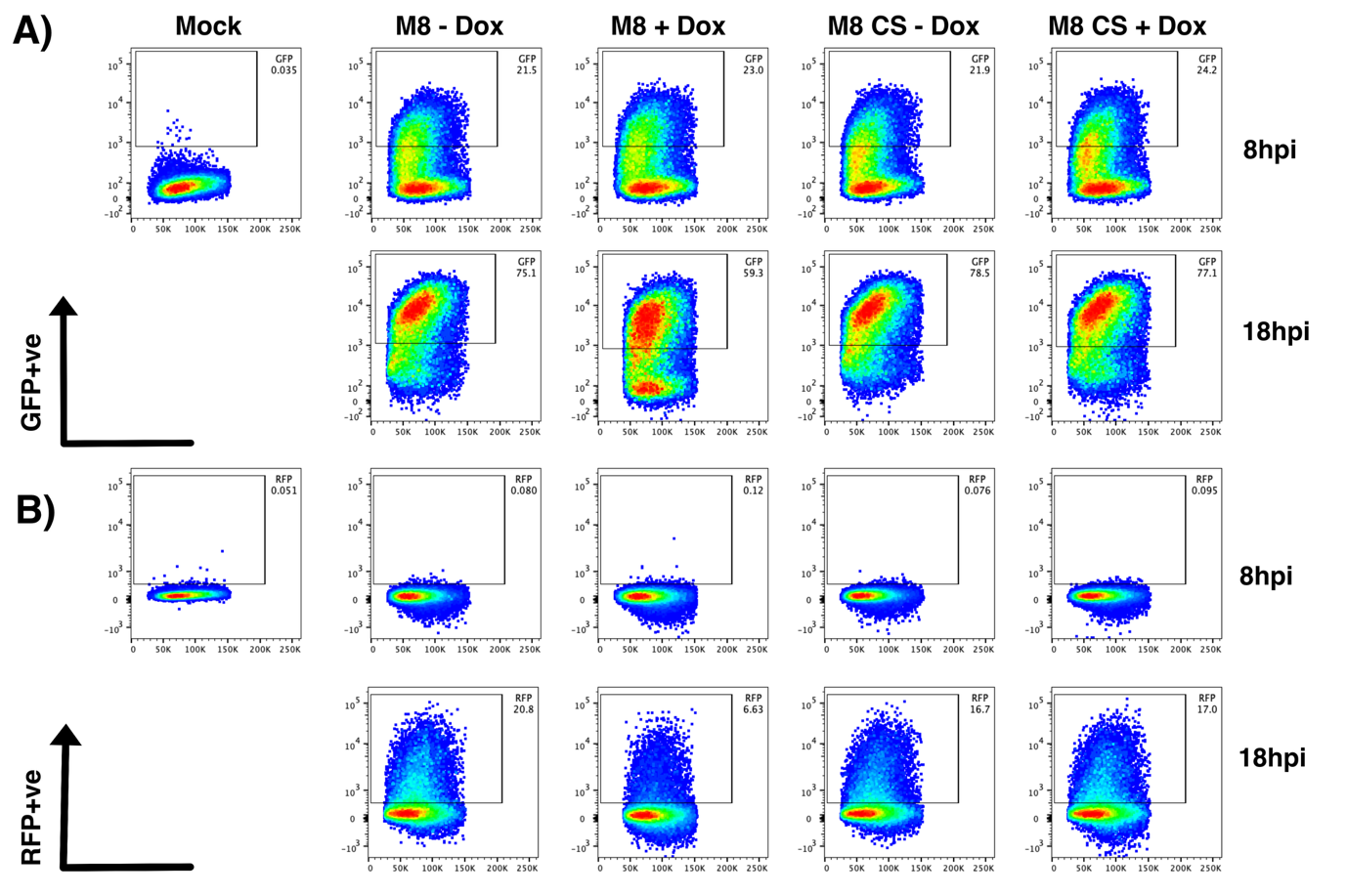


**Figure S5: MARCHF8 inhibit HSV-1 genomic replication and late viral gene expression.** 293T cells with Dox-inducible expression of FLAG-tagged M8 or M8-CS were cultured in the presence (Dox) or absence (No Dox) of 1ug/mL Dox for 24 hr (solid line) and then infected infected with a double fluorescent HSV-1 virus (DF-HSV-1), expressing GFP and RFP under control of the HSV-1 gB or gC promoters, respectively. At either 8 (MOI =2) or 18 (MOI = 5) hpi, cells were fixed and GFP (**A)** and RFP (**A)** expression analysed by flow cytometry. Representative dot plots are shown.


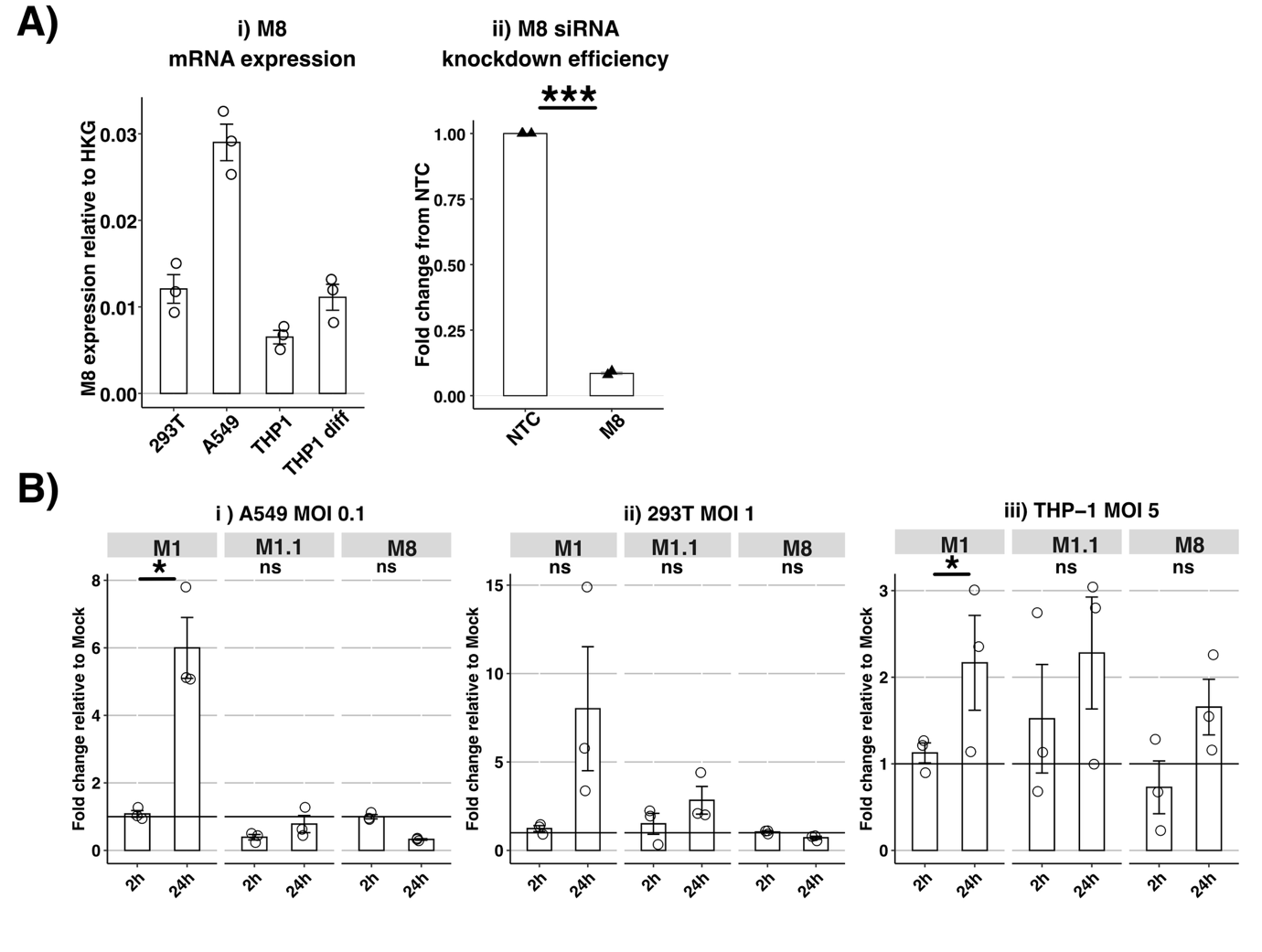


**Figure S6: MARCHF8 is efficiently knocked down in A549 cells and is not upregulated following HSV-1 infection. A) (i)** Expression of endogenous M8 in 293T, A549 and undifferentiated and PMA-differentiated THP-1 was determined by qPCR and expressed relative to housekeeping gene (GAPDH) (left panel). Technical replicates from 1 of 2 independent experiments are shown **(ii)** A549 cells were transfected with 10uM of M8-specific (M8) or non-targeting control (NTC) siRNA for 48 hr, and knockdown efficiency was determined by qPCR. Pooled data from three independent replicates (each done in singlicate) is shown. **B)** A549, 293T and THP-1 cells were either mock treated or infected with HSV-1 KOS (MOI 0.1, 1 or 5) and whole cell lysates were collected 2 or 24 hpi. Total RNA was extracted and mRNA levels of M8, M1 or M1.1 was determined by qPCR and expression levels relative to mock (after normalising to GAPDH expression) are shown. Technical replicates from 1 of 3 independent experiments are shown. Statistical analysis was performed using a mixed effects model utilizing data points from all experiments. * P<0.01, ** P<0.001, *** P<0.0001 and ns= not significant.


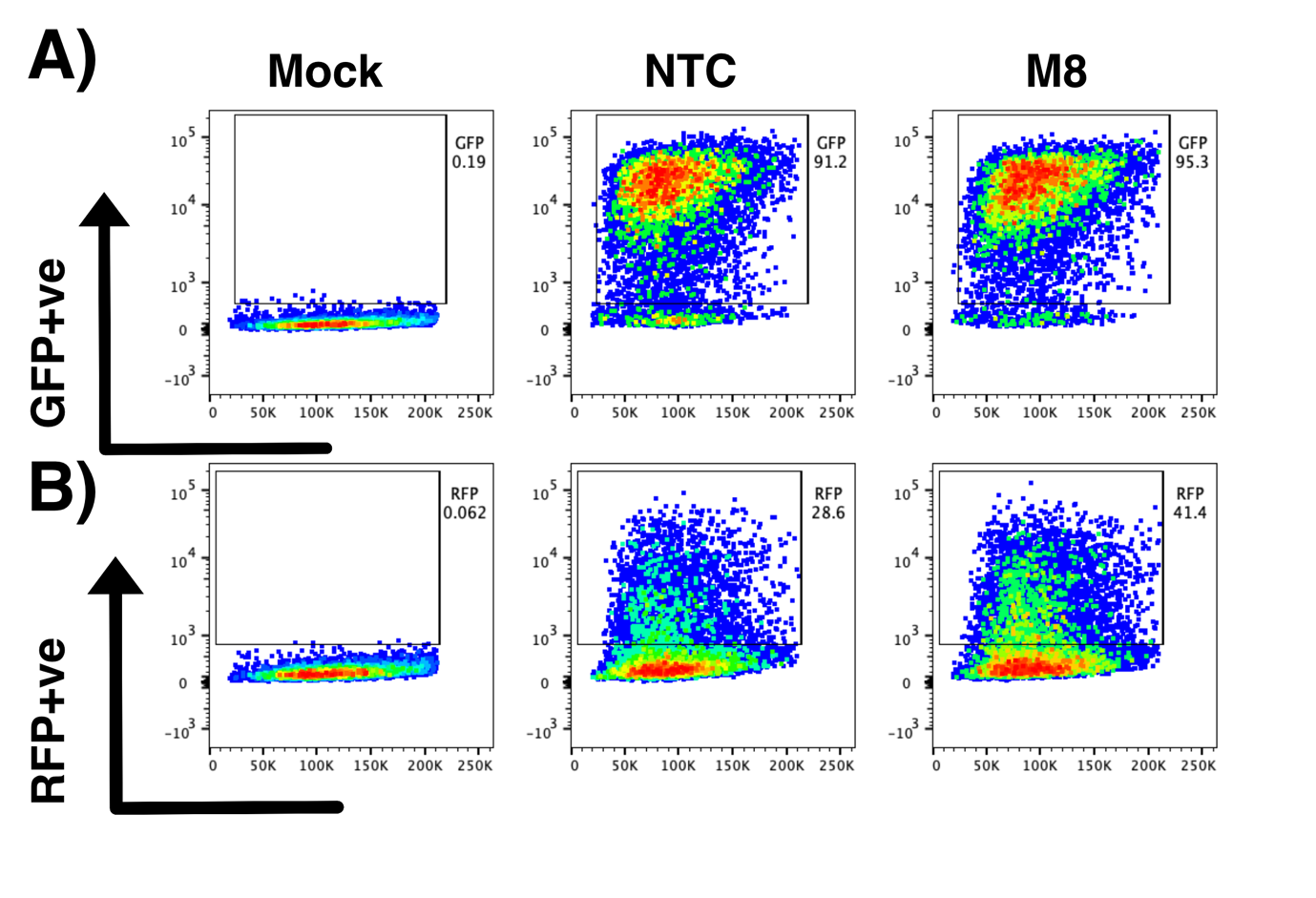


**Figure S7: MARCHF8 knockdown enhances GFP and RFP expression.** A549 cells were transfected with 10uM of M8-specific or NTC siRNA for 48 hr, infected with DF-HSV-1 (MOI 1) and levels of GFP or RFP expression determined by flow cytometry at 18 hpi. Representative dot plots for GFP **(A)** and RFP **(B)** are shown.

Reference:

1. Villalón-Letelier, F.; Farrukee, R.; Londrigan, S.L.; Brooks, A.G.; Reading, P.C. Isoforms of Human MARCH1 Differ in Ability to Restrict Influenza A Viruses Due to Differences in Their N Terminal Cytoplasmic Domain. *Viruses* **2022**, *14*, 2549, doi:10.3390/v14112549.
